# Rapid Collection and Aptamer-Based Sensitive Electrochemical Detection of Soybean Rust Fungi Airborne Urediniospores

**DOI:** 10.1021/acssensors.0c02452

**Published:** 2021-01-28

**Authors:** Vadim Krivitsky, Eran Granot, Yoav Avidor, Ella Borberg, Ralf T. Voegele, Fernando Patolsky

**Affiliations:** † School of Chemistry, Faculty of Exact Sciences, Tel Aviv University, Tel Aviv 69978, Israel; ‡ ADAMA Ltd., Tel Aviv 7015103, Israel; § Institute of Phytomedicine, University of Hohenheim, Stuttgart 70599, Germany; ∥ Department of Materials Science and Engineering, the Iby and Aladar Fleischman Faculty of Engineering, Tel Aviv University, Tel Aviv 69978, Israel

**Keywords:** electrochemistry, aptamers, spores, fungi, enzyme

## Abstract

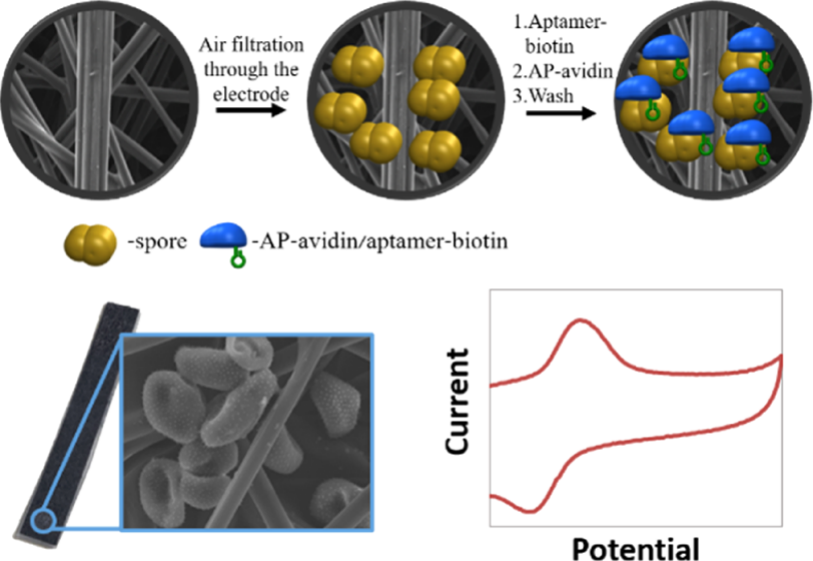

Plants are the central source of food for humans around the world. Unfortunately, plants can be negatively affected by diverse kinds of diseases that are responsible for major economic losses worldwide. Thus, monitoring plant health and early detection of pathogens are essential to reduce disease spread and facilitate effective management practices. Various detection approaches are currently practiced. These methods mainly include visual inspection and laboratory tests. Nonetheless, these methods are labor-intensive, time-consuming, expensive, and inefficient in the early stages of infection. Thus, it is extremely important to detect diseases at the early stages of the epidemic. Here, we would like to present a fast, sensitive, and reliable electrochemical sensing platform for the detection of airborne soybean rust spores. The suspected airborne soybean rust spores are first collected and trapped inside a carbon 3D electrode matrix by high-capacity air-sampling means. Then, a specific biotinylated aptamer, suitable to target specific sites of soybean rust spores is applied. This aptamer agent binds to the surface of the collected spores on the electrode. Finally, spore-bound aptamer units are incubated with a streptavidin–alkaline phosphatase agent leading to the enzymatic formation of *p*-nitrophenol, which is characterized by its unique electrochemical properties. Our method allows for the rapid (ca. 2 min), selective, and sensitive collection and detection of soybean rust spores (down to the limit of 100–200 collected spores per cm^2^ of electrode area). This method could be further optimized for its sensitivity and applied to the future multiplex early detection of various airborne plant diseases.

Multiple reasons exist requiring the detection of plant diseases. Knowledge about the presence of a disease is critically important for rapid treatment decisions, as it is closely related to yield and economic losses. Plants are the central source of food for humans around the world and create a balance between humans and their environment.^1,2^

Nevertheless, during their cultivation, plants can be negatively affected by diverse kinds of diseases. These plant diseases are responsible for major economic losses in the agricultural industry worldwide. So far, several studies reported the existence of more than 50,000 parasitic diseases, diseases occurring in plants due to the attack by an organism known as a parasite, and non-parasitic plant diseases in the United States.^3^

These diseases cause great damage to crops and therefore endanger food supply.^4^ For example, a huge amount of rice that can feed about 60 million people is destroyed by the blast disease every year.^5^

Furthermore, a critical aspect of crop diseases is their spreading mechanism. For instance, the potato late blight by *Phytophthora* infestans, which appeared at different locations in Europe during the 1845 epidemic, advanced exponentially with time, and the epidemic velocity increased linearly with distance.^6^

Thus, monitoring plant health and early detection of pathogens are essential requirements to reduce disease spread and facilitate effective management practices. In this context, various detection approaches are currently practiced. These methods mainly include visual inspection and laboratory tests. Visual inspection involves the identification of infected plants based on the presence of pathological symptoms. This approach is capable of detecting disease distribution within a wide range of field.^7^ Nonetheless, this method is labor-intensive, time-consuming, inefficient, and expensive in the early stages of infection.^8−12^ On the other hand, laboratory-based methods for the detection of plant diseases include physiological, biological, serological, and molecular tests.^13−16^ The most common laboratory tests are serological tests, such as the enzyme-linked immunosorbent assay, based on the use of protein in the detection of diseases, and also molecular tests, such as polymerase chain reaction (PCR) used in detecting plant diseases based on the DNA sequence of the pathogen.^17,18^ Although serological and DNA-based approaches have revolutionized plant disease detection; they are not very reliable at asymptomatic disease stages. This is due to the complexity of the methods and the time required for their performance.^19,20^ In addition, most of these DNA-based techniques are expensive and lack the rapidity required for the detection of plant diseases. Most techniques need at least 1–2 days for sample harvest, processing, and analysis. Additionally, none of these approaches is implementable in field, thus, it is extremely important to detect the disease at the early stages of the epidemic to improve disease control.^21,22^ Recently, the loop-mediated isothermal amplification technology was shown to be an important player in fungi spore’ detection. Nevertheless, this technique is limited by a higher risk of carry-over contaminations and more intricate assay designs, as it requires up to six primers, while PCR only requires two.^23^ Nevertheless, this approach has been recently successfully evaluated in field-detection applications.^24^

Specific and rapid detection assays for urediniospores could be a useful supplement to the time-consuming field monitoring of leaves for signs of pathogens and symptoms of disease development. The rapid and accurate identification of these diseases would improve the efficiency of fungicide applications by timing the use of fungicide sprays.

Specifically, soybean is one of the most common species of legumes, characterized by high nutritional values, and used as the main source of food for a vast population worldwide.^25−30^ Also, soybean is used as a food source for animals and as a raw material in the industry for the production of various products, such as glue and soap.^29−31^ Unfortunately, soybean crops are tremendously affected by rust diseases. Rust diseases are caused by pathogenic fungi (rust fungi), which can grow and reproduce only in living plants.^32−36^

There are about 5000 species of rust fungi that cause diseases on many types of plants, including *Phakopsora pachyrhizi*, *Puccinia boroniae*, *Phakopsora meibomiae*, and so forth. The rust disease is characterized by spots and blisters of orange-brown (rust) color on leaves, cankers, and galls on branches.^32−34^ The life cycle of rust diseases involves the production of spores. The rust spores can be easily and rapidly dispersed around enormous areas by the wind and infect entire fields in a matter of days. The spores can also be easily attached to animals, which enable them to spread the diseases to farther distances. Thus, sensing the soybean rust spores at the early stages is essential to avoid the infection of large areas of crops.

Soybean rust spores are characterized by an elliptical–spherical shape, reported to be in the size of (16.4–28.8) μm × (10.5–16.6) μm, and contain sharp thorn-like edges.^35^ It is possible to estimate the origin of spores by analyzing their morphology, but an accurate, reliable, fast, and selective sensing method is definitely required.^36^

Recently, there is growing interest in the development of electrochemical sensing of pathogenic spores.^37−40^ For example, Ait Lahcen et al. developed an electrochemical method for the sensing of *Bacillus cereus* spores based on spore-imprinted polymers.^37^ The sensor was developed using a carbon paste electrode, functionalized with an imprinted polymer, fabricated by pyrrole electropolymerization. The spore sensing was performed by recording cyclic voltammetry curves with the use of a redox probe. Also, in order to achieve the selective and specific detection of biomolecules, various nucleic-acid aptamers (single-stranded DNA or short RNA sequences) were developed.^38,41−43^ In this context, specific thiolated DNA-based aptamers against *B. cereus* spores were developed^38^ and used as specific recognition elements for electrochemical detection. The DNA-aptamer was linked to an Au electrode that traps *B. cereus* spores onto its surface, and the sensing of the spores was performed by AC impedance measurements with the use of redox probes. These electrochemical approaches have shown the capability to detect the pathogenic spores but unfortunately lack either the selectivity, sensitivity, on-field applicability, detection speed, or collection capability to be applied in the early diagnosis of plant diseases.^44^

In order to increase the sensitivity of airborne spores’ detection, without using complex and time-consuming steps, a few strategies could be considered. First, in order to serve as a sensor, the working electrode has to be composed of a material that can be easily chemically modified. Second, for air-phase analysis, the electrode should be used simultaneously as an air-collector filtering and a sensing agent. Additionally, for increased sensitivity and improved signal/noise ratio, the active surface of the working electrode has to be optimized. Carbon is well known for its diversified chemistry, thus makes an attractive sensor material.^45^ Furthermore, when composed of carbon microfibers (μCFs), it allows combining high conductivity, relatively low background currents, and high analytical signal.^46^ These μCF electrodes display high air permeability and allow free flow of air, thus potentially serving as air-sampling elements for the collection and adsorption of airborne species of interest. Additionally, these μCF electrodes display enormously high active surface areas, 1000–2500 m^2^/g, hence serve as highly effective elements for the improved adsorption of airborne species through air sampling means.^47,48^ Carbon paper electrodes (CPE) were used as an air diffusion layer for proton-exchange membrane fuel cells.^49^ The carbon paper surface can be modified with various materials and has been applied as the catalytic electrode for various types of fuel cell devices^50−52^ and for various types of electrochemical sensor devices.^53−56^ Thus, μCFs integrated as working electrodes in electrochemical sensors may enable the development of low-cost and sensitive sensors for airborne soybean rust spores.

Aptamers (DNA, RNA, or peptide) are examples of functional molecules selected in vitro. Aptamers are also termed “chemical antibodies” and prepared in vitro based on systematic evolution of ligands by exponential enrichment (SELEX). Unlike the preparation of antibodies, which rely on induction of an animal immune system, the SELEX process enables the synthesis of aptamers for non-immunogenic and toxic targets that are otherwise impossible to obtain by the immune system.^57^

Until now, aptamers have been selected toward a broad range of targets, including the metal ions, small organic molecules (e.g., amino acids, ATP, antibiotics, vitamins, and so forth) organic dyes, peptides and proteins, and even whole cells or microorganisms (e.g., bacteria). Importantly, the availability of such a large pool of aptamers makes it possible to develop novel bioassay tools covering areas that include diagnostics, anti-bioterrorism, and environmental and food analyses. Aptamers often possess high selectivity and affinity toward their targets. In addition to this high selectivity, aptamers bind to their targets with high affinity, particularly with macromolecules, which often possess remarkable dissociation constants (*K*_d_) ranging from picomolar to nanomolar.^58^ In particular, aptamer-based biosensors possess unprecedented advantages compared to biosensors using natural receptors such as antibodies and enzymes: first, aptamers with high specificity and affinity can in principle be selected in vitro for any given target, ranging from small molecules to large proteins and even cells, thus making it possible to develop a wide range of aptamer-based biosensors. Second, aptamers, once selected, can be synthesized with high reproducibility and purity from commercial sources. Also, in contrast to protein-based antibodies or enzymes, DNA aptamers are highly chemically stable. Third, aptamers often undergo significant conformational changes upon target binding. This offers great flexibility in the design of novel biosensors with high detection sensitivity and selectivity.^58^

Finally, incubation of the electrode in the presence of *p*-nitrophenyl phosphate non-electroactive reactant leads to the formation of electroactive *p*-nitrophenol in the vicinity of the electrode surface, thus allowing the selective amplified detection of spores by electrochemical means. Notably, only the collection of specific airborne spores will lead to the formation of the electroactive product, *p*-nitrophenol, and to the electrochemical sensing signal.

Here, we would like to present a fast, sensitive, and reliable electrochemical sensing platform for the detection of airborne soybean rust spores. The suspected airborne soybean rust spores are first collected and trapped inside the carbon 3D electrode matrix by high-capacity air-sampling means, 30–100 L/min. Then, a specific biotinylated aptamer developed by us, suitable to target specific sites of soybean rust spores is applied. This specific aptamer agent solely binds to the surface of the collected spores on the electrode, serving as a first amplification step through the binding of multiple aptamer units per adsorbed spore. Finally, spore-bound aptamer units are incubated with a streptavidin–alkaline phosphatase (AP) reporting agent leading to the second amplification step by enzymatic formation of the electroactive product *p*-nitrophenol, which is directly characterized by its unique electrochemical properties.^59,60^ The electrochemical properties of *p*-nitrophenol were studied with the use of various types of electrodes.^59−64^ Under the presented experimental conditions, only the soybean rust spores collected on the electrode bind to the specific biotinylated aptamer units, which are linked to the streptavidin–AP couple, and therefore, the *p*-nitrophenol enzymatic product only forms as a result of the presence of soybean rust spores. Our method allows for rapid (ca. 2 min), selective, and sensitive collection and detection of airborne soybean rust spores (down to the limit of 100–200 collected spores per cm^2^ of electrode area). This method could be further optimized for its sensitivity and applied to the future multiplex early detection of various airborne plant diseases by simple electrochemical means.

## Experimental Section

Triple distilled water (TDW) from a Milli-Q (Millipore) source was used throughout the experiments. All salts and solvents were purchased from Merck and used without further purification. *P*-nitrophenyl phosphate, *p*-nitrophenol, *p*-aminophenyl phosphate, *p*-aminophenol, streptavidin–AP from *Streptomyces avidinii*, and bovine serum albumin (BSA powder) were purchased from Merck.

Aptamers with specific affinity against *P. pachyrhizi* soybean rust spores (100 nmol DNA oligo, 88 bases, HPLC-purified, 5′ biotin) were developed with the services of Novaptech Ltd. (France, see Supporting Information Materials Section for details on aptamer development) and purchased from Syntezza Bioscience Ltd.

The aptamer sequence (modified with biotin at 5′) is 5′-AGCCTGTTGTGAGCCTCCTGTCGAATATGGGGTGGGTGGGTGGCATTTGAAGG GGCTCGCACACTTTGAGCGTTTATTCTTGTCTCCC-3′.

Soybean rust spores are the *P. pachyrhizi* isolate Thai 1 from the collection of the laboratory of Prof. Ralf Thomas Voegele (University of Hohenheim, Stuttgart, Germany). This isolate goes back to a single spore isolate obtained from Thailand. Also, *P. pachyrhizi* urediniospores from naturally infected soybean plants were obtained from the Adama Brazil Ltd. collection, Brazil. The spores collected were germinated in sterile deionized water for 24 h at 22 °C. After centrifugation at 7000 xg for 10 min, the pellet was suspended in PBS and frozen at −20 °C. Also, closely related *P. meibomiae* urediniospores used as negative control were received from the collection of Prof. Ralf Thomas Voegele (University of Hohenheim, Stuttgart, Germany). All spore concentrations were determined by the use of hemocytometry.

Carbon paper electrodes (CPEs, 254 μm thick) that contain multi-layers of μCFs (type Spectracarb 2050A-1050) were purchased from Engineered Fibers Technology. Commercial Ag/AgCl (sat. KCl) reference electrodes (type RE-1CP) were purchased from ALS Ltd. Pt (99.999%, 1 mm diameter) was used as a counter electrode, purchased from Holland-Moran Ltd.

The electrochemical experiments were performed by a potentiostat (EmStat,^3^ PalmSense) with the use of PSTrace 5.6 software.

Two successive cyclic voltammetry (CV) cycles were performed (starting at 0 V vs reference) for each measurement and the second CV curve is presented.

The soybean rust spores on the CPE were imaged using a light microscope (Olympus BX41m-LED with the use of a U-PMTCV camera adapter in a dark-field mode). The soybean rust spores on CPE were sputtered (4 nm Au/Pd, Emitech SC7640 sputter coater, Polaron) and imaged using Quanta 200 FEG ESEM (Thermo Scientific) in a high-vacuum mode.

### Aptamer Selection Sequence (for the Complete Aptamer Production Procedure, See the Supporting Information Materials Section)

Phase 1: Screening of DNA library.

1 Selection (positive) at room temperature in a 100 mM carbonate buffer pH = 9.5 containing 10 mM sodium chloride, 40 mM potassium chloride, and 5 mM magnesium chloride hexahydrate, by filter retention of candidates from the library in the presence of *P. pachyrhizi* urediniospores.
2 Negative selection under similar conditions against *P. meibomiae* urediniospores; PCR amplification of the selected pool; production of single-stranded candidates for the next selection round.
3. Selection (positive and negative) over six–eight rounds under increasing stringency.
4. Evaluation of average binding properties of the selected pools.

Phase 2: selection of polyclonal aptamers to *P. pachyrhizi* urediniospores.

1. Selection (positive and negative) of candidates using a process identical to that of phase 1, starting from a pool of phase 1 (a single condition).
2. Selection over four–six additional rounds under increasing stringency.
3. Production of 8–10 pools with indexed primers for NGS sequencing.
4. NGS sequencing (105–106 reads per round).
5. Bioinformatic analysis of the sequences (primary sequences, predicted secondary motifs, and round-to-round evolution).
6 Definition of clusters.
7. Identification of 10–15 sequences from bioinformatics.

Phase 3: identification and characterization of monoclonal aptamer(s) to *P. pachyrhizi* urediniospores.

1. Synthesis of 10–15 candidates identified in phase 2.
2. Evaluation of binding properties of these 10–15 candidates to *P. pachyrhizi* urediniospores by fluorescence.
3. Evaluation of specificity of these candidates against *P. meibomiae* urediniospores.
4. Identification of the best candidate(s).

Phase 4: optimization of the best aptamer to *P. pachyrhizi* urediniospores.

1. Truncation of the best aptamer (1 aptamer): synthesis of five variants at most.
2. Evaluation of binding properties of the five variants to *P. pachyrhizi* urediniospores by fluorescence.
3. Definition of the best variant.

*K*_d_ of the positive aptamer was estimated experimentally to be ca. 18 nM.

### Electrochemical Sensing Setup and Detection Procedure

0.1 M carbonate buffer solution (CBS) containing 0.005 M MgCl_2_ and 0.01 M NaCl, pH 9.0 was used in all experiments, including for the development of specific aptamer units.

The carbon paper was cut into rectangular pieces of 7 × 50 mm, followed by polyethylene lamination. The active (non-laminated) area of the CPE was designed to be 4 mm diameter.

Each CPE was washed twice with TDW and isopropanol and soaked in two BSA buffer solutions (5mg·mL^–1^, 1:1 v/v TDW/CBS), 10 min each, then the surface-blocked CPE (CPE/BSA) was rinsed three times with CBS and dried gently by a N_2_ stream.

**Figure 1:**
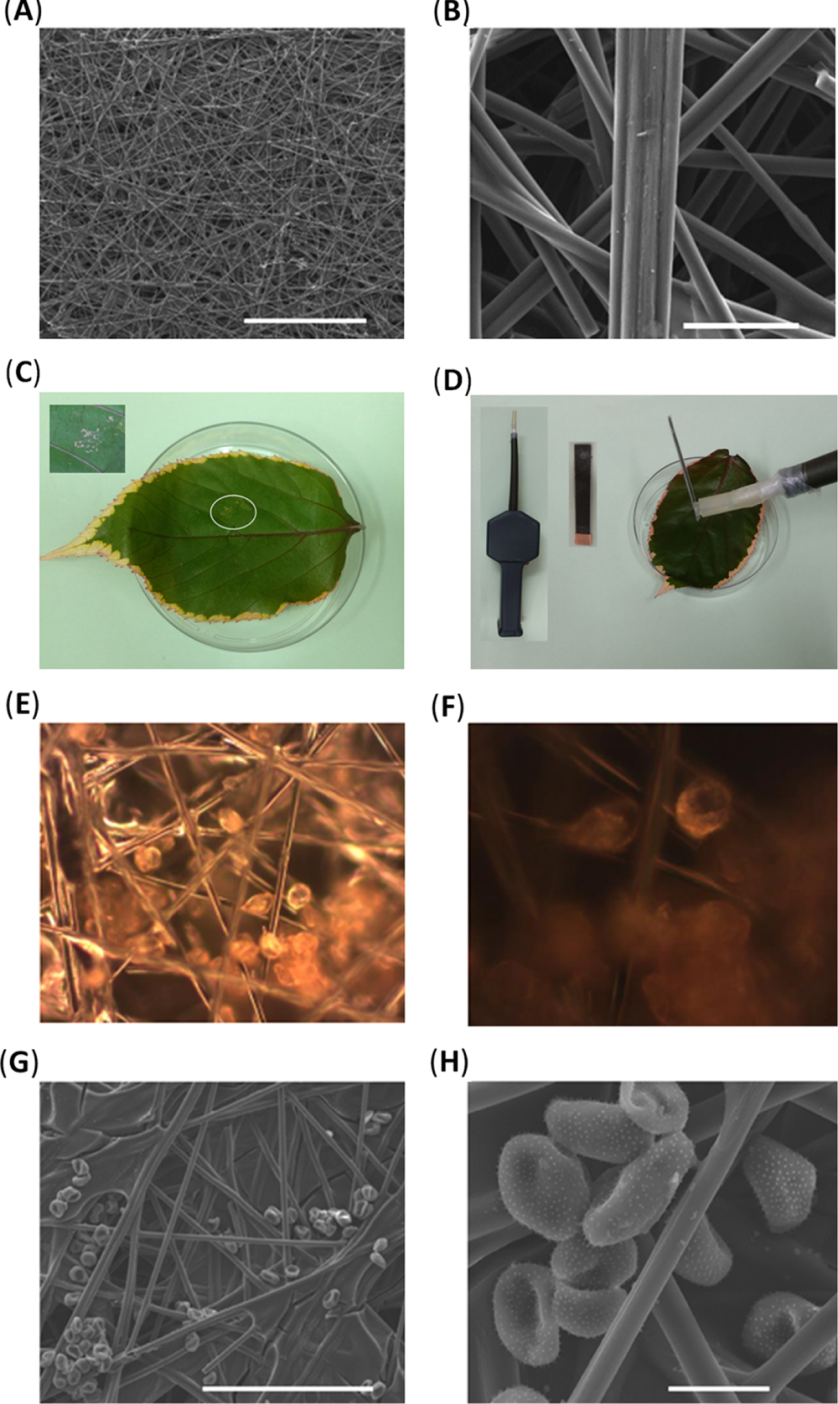
SEM images of our CPE. Scale bars are 1 mm for (A) and 50 μm for (B). SEM images were performed by the secondary electron mode (20 kV, 10 mm). The collection process of the *P. pachyrhizi* spores on the surface of the CPE. (C) Soybean rust spores quantitatively dispersed on the leaf sample. (C) Inset: Expanded area of the rust spores dispersed on the leaf sample. (D) Collection process of soybean rust spores from the leaf to the CPE by our home-made air sampler. (D) Inset: Our home-made laminated CPE and our home-made air sampler. Light microscopy dark-field images of *P. pachyrhizi* soybean rust spores collected on CPE at various magnifications: (E) ×20 and (F) ×50, after 3 min of sampling the surrounding of soybean rust sample with the use of our home-made air sampling system, at a pace of 10 L·min^–1^. SEM images of sputtered (Au/Pd, 4 nm) *P. pachyrhizi* soybean rust spores collected on the CPEs. Scale bars are 200 μm for (G) and 20 μm for (H) SEM images were obtained by the secondary electron mode (20 kV).

The soybean *P. pachyrhizi* rust spore suspension containing a known concentration, 10^2^ to 10^4^ spores, were drop-casted on top of a *Viburnum sieboldii* leaf to simulate a real-world collection scenario, and further transferred to the collecting CPE by pumping the air at a 10 cm distance from the leaf, using a home-made air sampler shown in Figure 1D (30–100 L/min capacity). Our sampler device includes a hose and a pump. Also, to determine the detection limit of our approach, the soybean rust spores at a given amount, 10^2^ to 10^4^ spores per 10 μL, were directly dispersed on top of the collecting CPE using a pipette, followed by the electrochemical detection of spores as per the given procedure. A detection limit of ca. 100–200 spores can be reliably achieved (*n* > 30 repetitions at this detection limit concentration).

Then, the spore-embedded CPE electrodes were incubated with the biotinylated aptamer, which is suitable to target specific sites of soybean rust spores (1 μL solution of 100 μM, 60 s), followed by washing with three CBSs, pH 9 (1 min for each solution). The 60 s period was selected to ensure the saturation of avidin–biotin coupling.^65−68^ Then, the CPE electrodes were incubated with streptavidin–AP (1 μg·μL^–1^ in CBS, 1 μL, 60 s), followed by washing with three CBSs, pH 9 (1minute for each solution). Similarly, CPE electrodes after the collection of non-specific disease spores were treated as described for control experiments.

The limit of detection of the method was extracted by measuring the resulting signal, after the whole described protocol of detection is performed and after deposition of a controlled number of spores on the center of the carbon electrodes. A sensitivity limit of ca. 100 spores was achieved for >20 measurements.

All the experiments presented in the paper were repeated at least 30 times (*n* > 30). For each set of experiments shown in the paper, at least 20 electrodes were used for repetitions.

## Results and Discussion

**Scheme 1:**
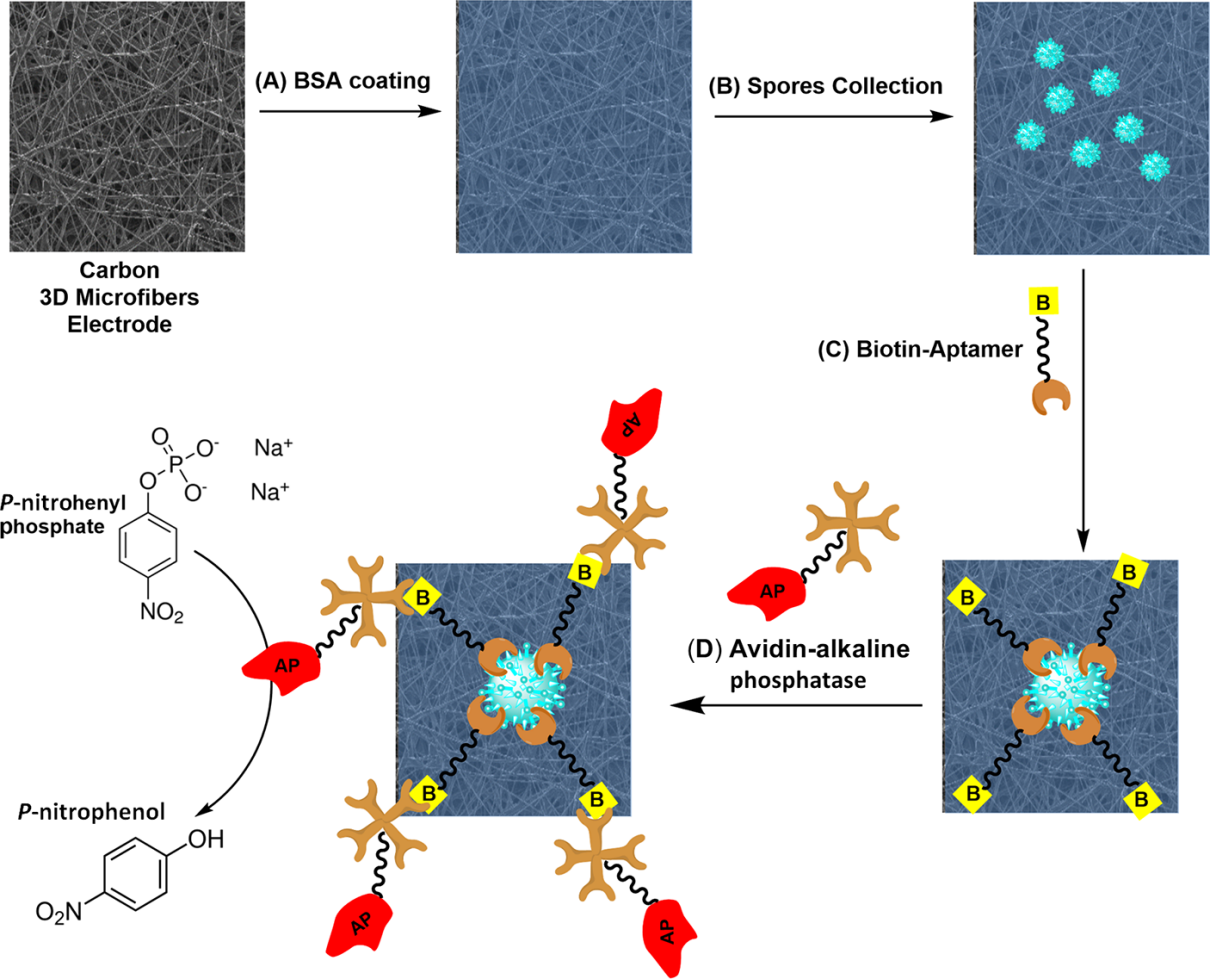
Schematic Description of the Collection and Detection Approach for Airborne Soybean Rust Spores; (A) BSA Blocking of the Carbon Fiber Electrode; (B) Spore Collection Step; (C) Biotin–Aptamer Binding Step. (D) Avidin–Enzyme Conjugate Binding and Enzymatic Amplification Reaction

The schematic procedure for the detection of airborne soybean rust spores is shown in Scheme 1. First, the surface of the CPE was blocked with BSA to avoid non-specific absorption of the aptamer and enzyme (A). The spores are collected by the electrode matrix through air-sampling means (B). This leads to spore-embedded electrodes. These electrodes are then incubated shortly with the specific biotinylated aptamer and the enzyme–avidin conjugate, (C) and (D), respectively, leading to the first amplification step of binding of multiple aptamer units to each single collected spore.

In order to develop a selective *P. pachyrhizi* soybean rust spore-sensing device, it is first essential to develop a method for collecting rust spores around the surface of the CPE. It is also essential to prevent the non-specific binding of aptamer and enzyme to the surface of the carbon electrodes, solely leading to their binding to the collected spore units. In addition, it is important to study the electrochemical behavior of *p*-nitrophenol under our experimental sensing conditions.

The μCF paper was used as a base working electrode due to its large area and high adsorption capacity. Figure 1A,B presents the SEM (scanning electron microscopy) images of our CPE. The diameter of the carbon fibers is about 5 μm.

In order to meet the requirement for a portable and robust electrochemical sensing system that can be applied under field conditions, a portable home-made air sampler was used. Our air sampler device includes a hose and a pump, capable of pumping 30–100 L·min^–1^. This air sampling device is powered by rechargeable batteries. The main purpose of the air sample system is to perform a collection of the soybean rust urediniospores on the electrode surface. Importantly, the electrode is fully air permeable, which enables its air-filtering capabilities.

Figure 1C,D presents the collection process of soybean rust spores on the surface of the CPE by air-collection means. The soybean rust spores are quantitatively dispersed on a leaf sample to simulate a real-world collection scenario (C), and then the soybean rust spores are collected (during 10 s) in the CPE by our home-made air sampler (D).

The soybean rust spores, embedded in the CPE, can be observed by light microscopy dark-field images, as presented in Figure 1E,F. Those images show that the soybean rust spores attach to the microfiber’ surface of the CPEs.

Figure 1G,H presents the SEM images of sputtered soybean rust spores embedded in the CPEs. These SEM images show the morphology of the *P. pachyrhizi* soybean rust spores. Both images show that the rust spores are also attached to the internal planes of the CPE.

**Figure 2:**
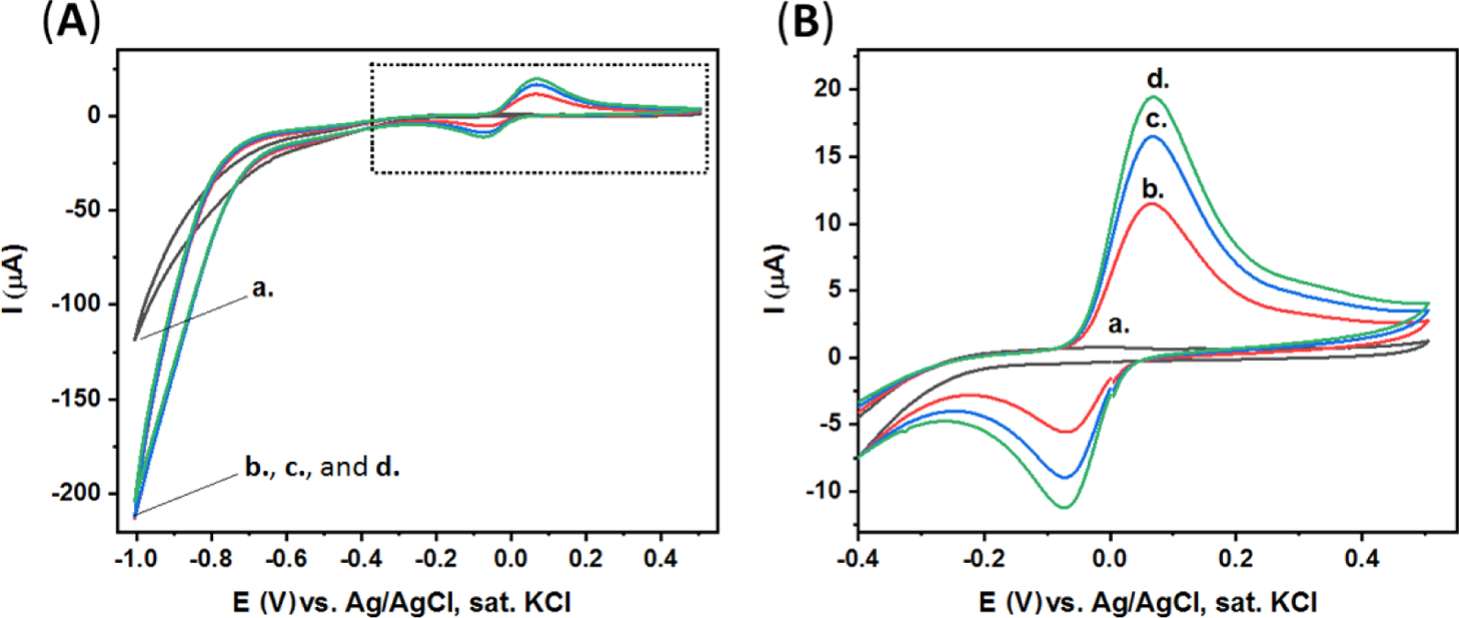
(A) CV of our clean CPEs modified with the aptamer and enzyme. Curve (a) in CBS. Curves (b–d) after the addition of *p*-nitrophenyl phosphate substrate (2 mg, 5 mL CBS) at different time intervals: 0, 5, and 10 min, respectively. (B) Expanded dotted area of (A). Scan rate, 100 mV·s^–1^.

The electrochemical properties of *p*-nitrophenol were studied with the use of various types of electrodes.^54−58^ Importantly, our studies, Figure 2, show that both the aptamer and enzyme strongly and non-specifically absorb to the surface of clean CPEs, resulting in the subsequent unwanted false *p*-nitrophenol (and *P. pachyrhizi* soybean rust spores) detection.

**Figure 3:**
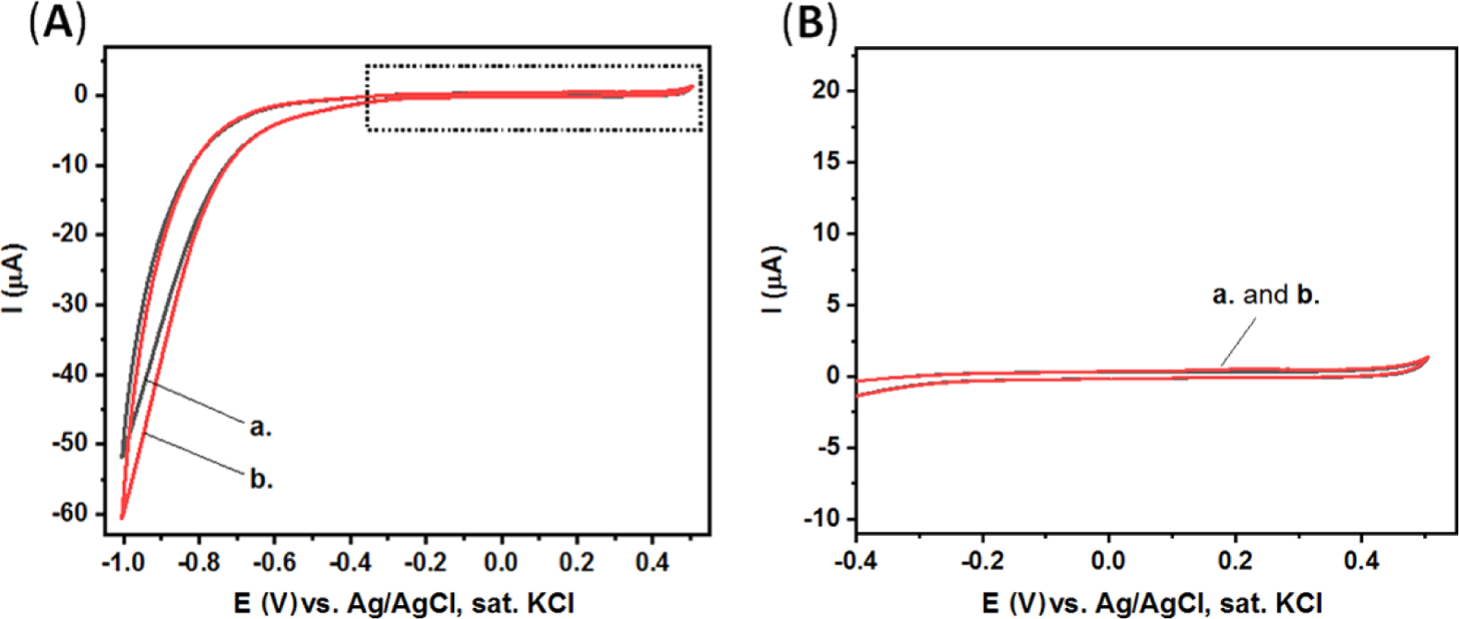
(A) CV of our CPE blocked with BSA (CPE/BSA) and modified with the aptamer and enzyme. Curve (a) in CBS. Curve (b) after the addition of *p*-nitrophenyl phosphate substrate (2 mg, 5 mL CBS). Scan rate, 100 mV·s^–1^. (B) Expanded dotted area of (A).

Thus, in order to solve this apparent challenging limitation of our high-surface electrodes, a blocking modification step is performed using BSA. BSA is used widely as a surface blocking agent to prevent the non-specific adsorption of proteins and DNA to the surfaces of sensing devices.^69−71^ In our study, BSA was found as a suitable additive that completely prevents the non-specific adsorption of aptamer and enzyme units to the surface of the CPEs, Figure 3. Evidently, the redox current generated by the CPE/BSA electrode is negligible (Figure 3) compared to the redox current generated by the clean CPE (Figure 2).

**Figure 4:**
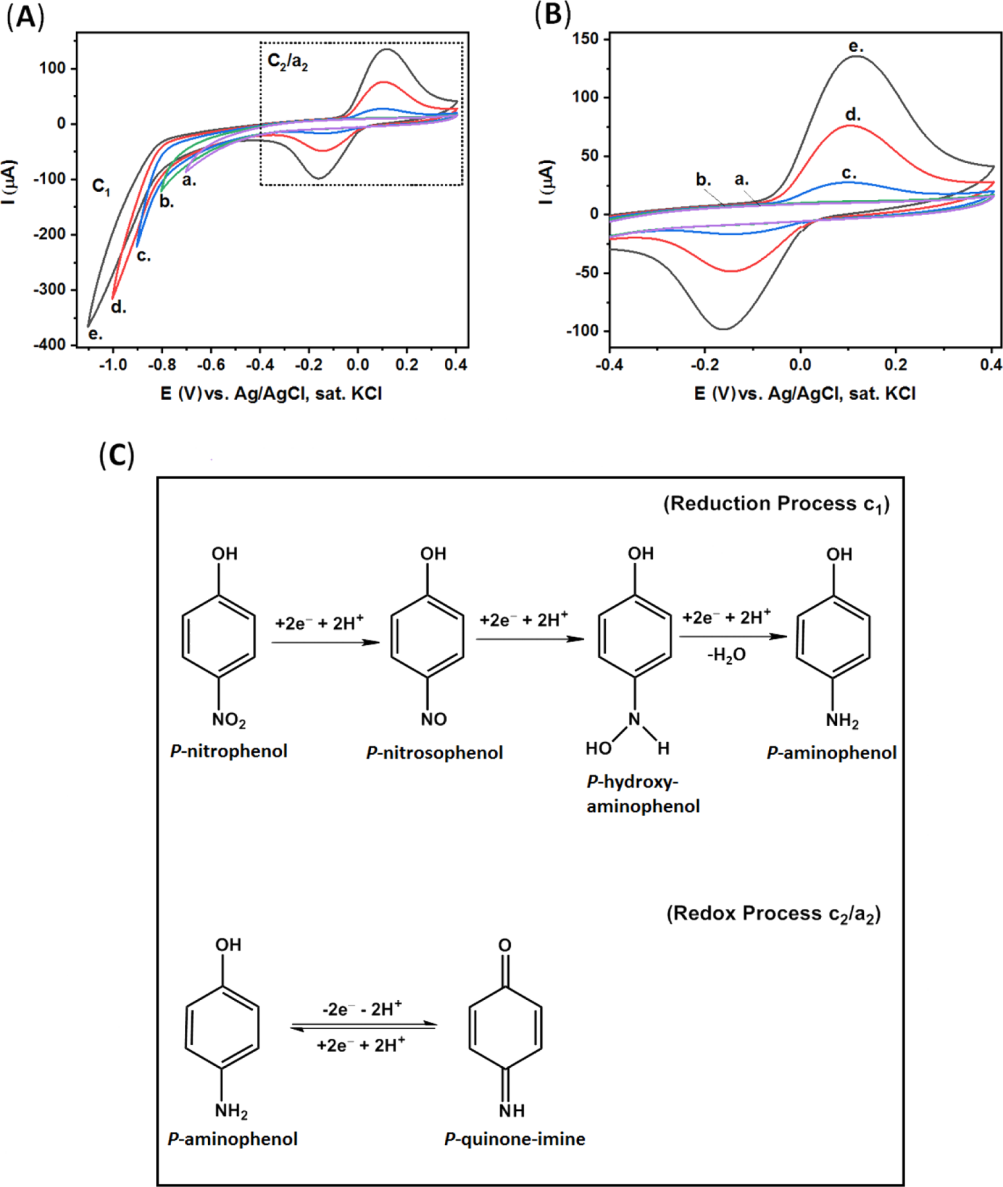
(A) CV of our CPE/BSA in *p*-nitrophenol solution (0.8 mM, CBS), when different negative potentials were applied: (a) −0.7, (b) −0.8, (c) −0.9, (d) −1.0, and (e) −1.1 V. Scan rate, 100 mV·s^–1^. (B) Expanded dotted area of (A). (C) Electrochemical reduction mechanism of *p*-nitrophenol.

Figure 4A presents the CV results of our CPE/BSA electrode in a *p*-nitrophenol solution, 0.8 mM in CBS, when different potential limits were applied. Figure 4B presents the expanded dotted area of (A). The electrochemical mechanism of *p*-nitrophenol includes two successive steps,^63,64^ as presented in Figure 4C; the first step is the irreversible reduction of *p*-nitrophenol (to *p*-aminophenol), *c*_1_, and the second step is the redox process of *p*-aminophenol (to *p*-quinone-imine), *c*_2_/*a*_2_.

In our CPE/BSA electrodes, the reduction of *p*-nitrophenol (to *p*-quinone-imine) does not occur in a negative potential of −0.7 V and up to −0.8 V versus Ag/AgCl (sat. KCl), curves (a,b), respectively. The irreversible reduction of *p*-nitrophenol (to *p*-quinone-imine), c_1_, occurs at potentials lower than −0.8 V, curve (c), resulting in the quasi-reversible reaction of *p*-quinone imine (to *p*-amino-phenol), *c*_2_/*a*_2_, appearing at ca. +0.11 V and ca. −0.16 V (B).

When the negative potential is increased to −1.0 V and −1.1 V, curve (d) and curve (e), respectively, significantly more *p*-nitrophenol is reduced, *c*_1_, and the redox peaks of *p*-quinone-imine dramatically increase, *c*_2_/*a*_2_. These results demonstrate the optimal voltage conditions required for the most sensitive detection to be allowed.

As mentioned before, the CPEs are surface-blocked with BSA, Scheme 1A, in order to prevent non-specific absorption of aptamer and enzyme to the CPE surface and then soybean rust spores are collected on the surface of the CPE/BSA electrodes by the use of a home-made air sampler (B). The CPE/BSA is then incubated in the presence of biotinylated aptamers with specific affinity toward soybean rust spores (C) and then incubated with streptavidin–AP (D) resulting in the formation of the biotin–avidin complex around the surface of the soybean rust spores.

Notably, steps (C) and (D) can be performed in a single step. *P*-nitrophenol is the electroactive enzymatic product of AP formed in the presence of an electrochemically inactive *p*-nitrophenol phosphate substrate. The detection of *p*-nitrophenol is evidence of the presence of collected *P. pachyrhizi* soybean rust spores on the CPEs.

Additionally, *p*-aminophenyl phosphate was tested as another substrate of the enzymatic reaction instead of the currently used *p*-nitrophenyl phosphate derivative, shown in Supporting Information Figures S16 and S17; the series of experiments revealed that both substrates display very similar performance in relation to the sensitivity of the assay. The only difference observed is the electrochemical window applied for sensing purposes.

It is important to emphasize that the CPE consists of multilayers of μCFs, thus the enzyme-modified soybean rust spores are also attached to the internal planes of the CPE. The *p*-nitrophenol product can also be formed in the internal planes of the CPEs and can also diffuse into even deeper planes of the CPEs, resulting in increased amperometric responses.

CPE/BSA electrodes that were not used for the air collection of specific spores and CPE/BSA electrodes after their incubation with the aptamer and enzyme conjugate (without the collection of spores) were tested as control experiments. CV measurements were performed immediately after each electrode has been soaked in *p*-nitrophenyl phosphate substrate solution. In addition, additional CV measurements (for each electrode) were performed at different time intervals, up to 15 min.

**Figure 5:**
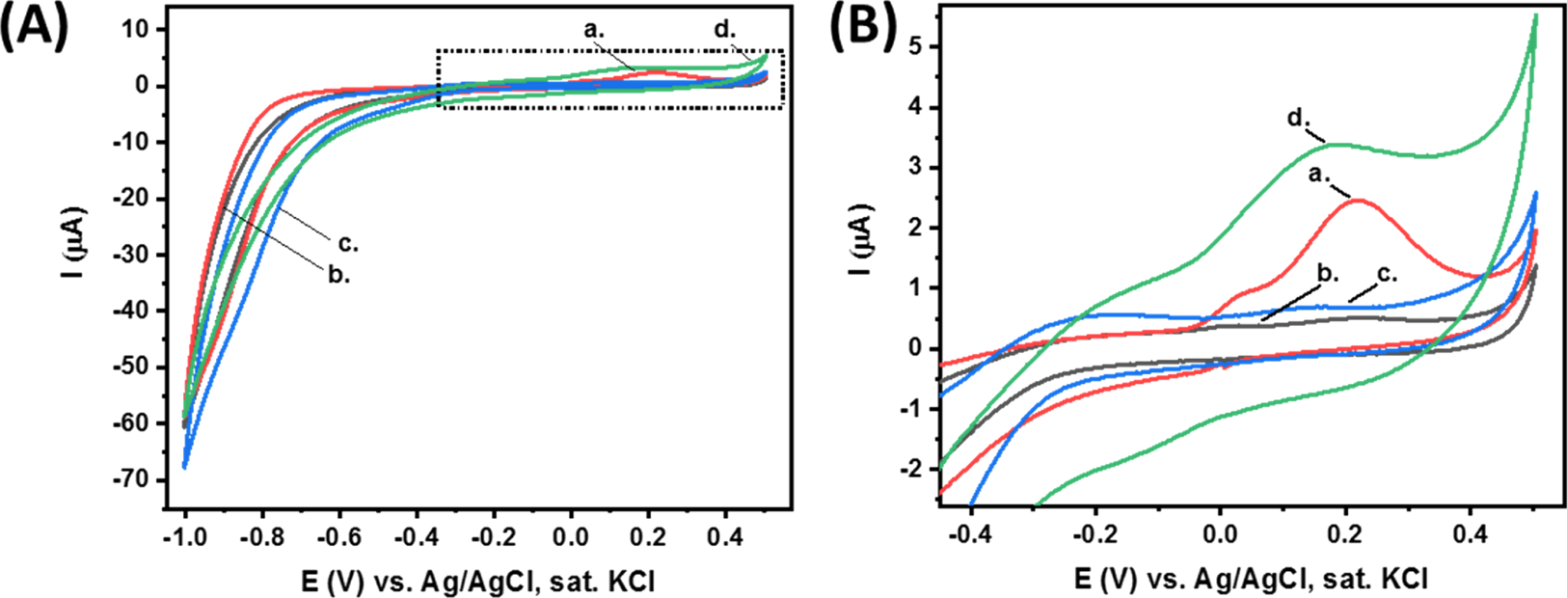
(A) CV of different CPE/BSA electrodes immediately after being soaked in *p*-nitrophenyl phosphate substrate solution (2 mg in CBS, 5 mL): Curve (a) fully modified CPE (containing the *P. pachyrhizi* soybean rust spores (German source), the aptamer, and the AP enzyme). Curve (b) CPE that does not include the soybean rust spores (modified only with aptamer and enzyme). Curve (c) clean CPE/BSA. Scan rate, 100 mV·s^–1^. Curve (d) Fully modified CPE (containing the *P. pachyrhizi* soybean rust spores (Brazilian-source), the aptamer, and the AP enzyme) (B) Expanded dotted area of (A).

Figure 5 presents the CV results of each electrode immediately after it has been soaked in the substrate solution. The redox peaks presented in these voltammograms are in accordance with the expected electrochemical properties of *p*-nitrophenol, as presented in Figure 4. These results demonstrate the specificity of the detection platform in the absence of collected spores. No evident electrochemical signals result from the detection sequence in this case. This is evidence that no aptamer or enzyme conjugate is adsorbed onto the electrode surface in the absence of specific spores, and no formation of the electroactive product nitrophenol occurs.

On the other hand, the collection of specific *P. pachyrhizi* soybean spores through air collection leads to the appearance of a clear electrochemical response for the *p*-nitrophenol enzymatic product. Evidently, the oxidation current at ca. +0.2 V generated by the fully modified electrode in the presence of *P. pachyrhizi* soybean spores (German and Brazilian source), curves (a,d), is ca. 10 times higher compared with the oxidation current generated by the control CPE/BSA electrode in the absence of soybean spores, curve (b), and with the oxidation current generated by the clean CPE/BSA, curve (c).

**Figure 6:**
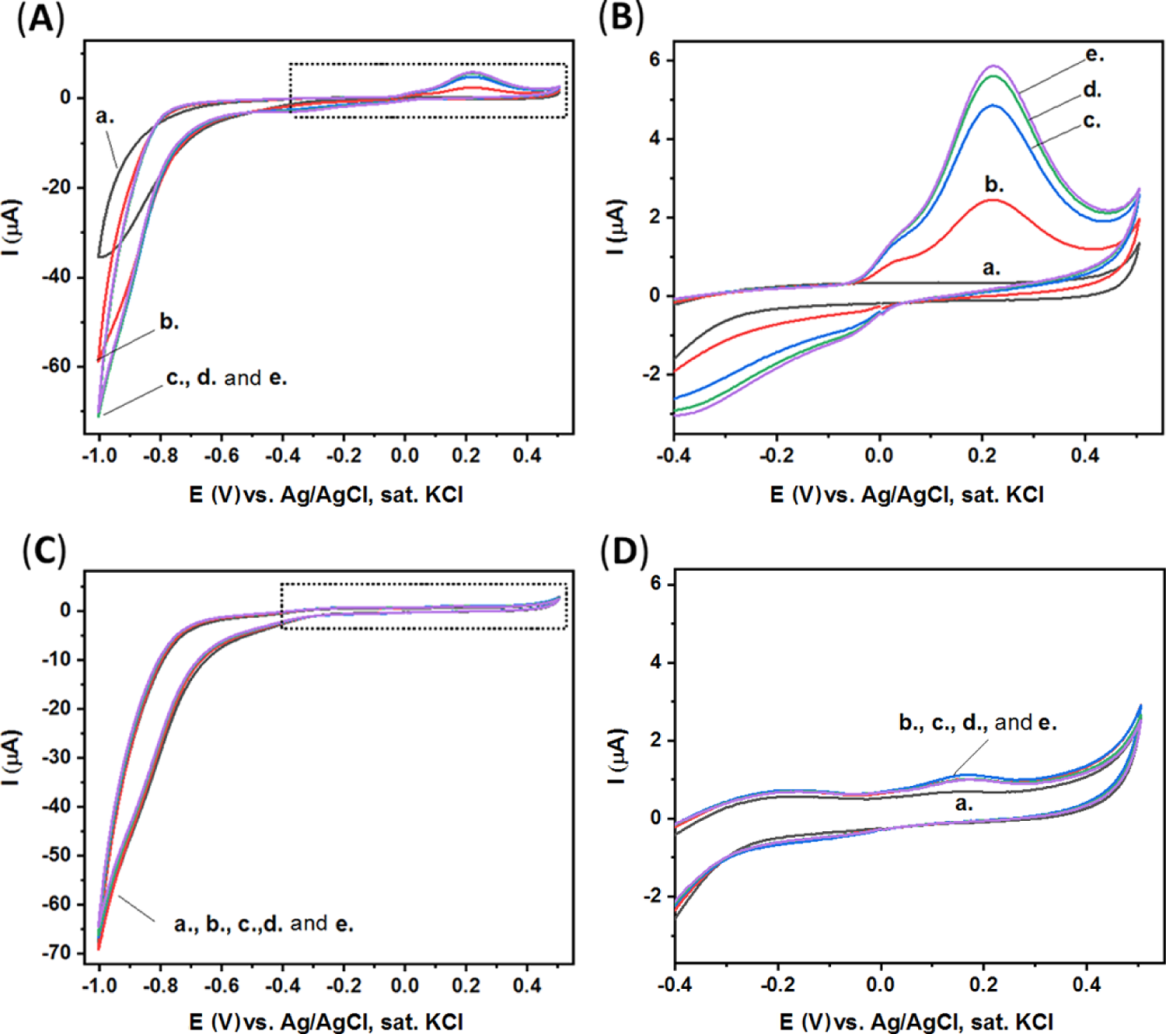
(A) CV of CPE/BSA fully modified, contains the *P. pachyrhizi* soybean rust spores, the aptamer, and the AP enzyme: curve (a) in CBS. Curves (b–e) after the addition of *p*-nitrophenyl phosphate substrate (2 mg in CBS, 5 mL) at different time intervals: 0, 5, 10, and 15 min, respectively. Scan rate, 100 mV·s^–1^. (B) Expanded dotted area of (A). (C) CV of CPE/BSA: curve a in CBS. Curves (be) after the addition of *p*-nitrophenyl phosphate substrate (2 mg in CBS, 5 mL) at different time intervals: 0, 5, 10, and 15 min, respectively. Scan rate, 100 mV·s^–1^. (D) Expanded dotted area of (C).

Figure 6A–D presents the CV curves of these three different electrodes for different time intervals, up to 15 min. Similarly, the oxidation current at ca. +0.2 V generated by the fully modified electrode, Figure 6A,B, is significantly higher compared with the oxidation current generated by the untreated CPE/BSA in the absence of soybean spores, Figure 6C,D.

It seems that *p*-nitrophenol is formed in minuscule amounts without the presence of AP enzyme, as presented in Figure 6C,D. Notably, the formation of *p*-nitrophenol is catalyzed dramatically by the AP enzyme attached specifically to the *P. pachyrhizi* soybean rust spores collected by the electrode, as presented in Figure 6A,B.

**Figure 7:**
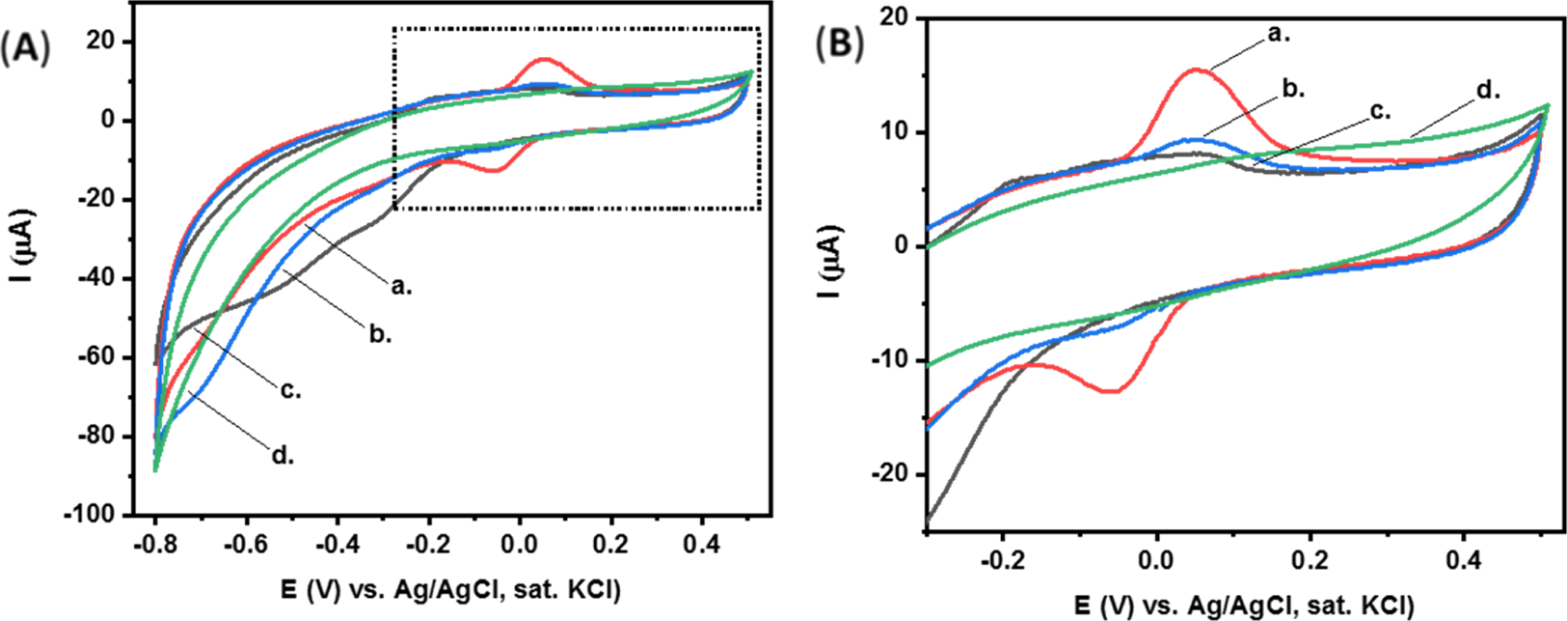
(A) CV of different CPE electrodes modified with BSA after air-sampling for 1 min at different atmospheres: curve (a) rust spores, curve (b) powdery mildew spores, curve (c) air lab, and curve (d) *P. meibomiae* spores. The CV measurements were performed in a solution containing *p*-nitrophenyl phosphate substrate (2 mg in CBS, 5 mL) after 1 min of incubation. Scan rate, 100 mV·s^–1^. (B) Ddotted area of (A).

Furthermore, control experiments conducted for the detection of additional non-specific spore species, powdery mildew and the closely related *P. meibomiae* spores, were performed, Figure 7A. These results demonstrate the clear-cut selectivity of our sensing platform against the specific urediniospores of interest. The redox current generated by the CPE/BSA consisting of the rust spores, curve a, is significantly higher compared to the CPE/BSA against the mildew and *P. meibomiae* spores and the clean CPE/BSA in the atmosphere of the air lab, curve (b), curve (d), and curve (c), respectively.

**Figure 8:**
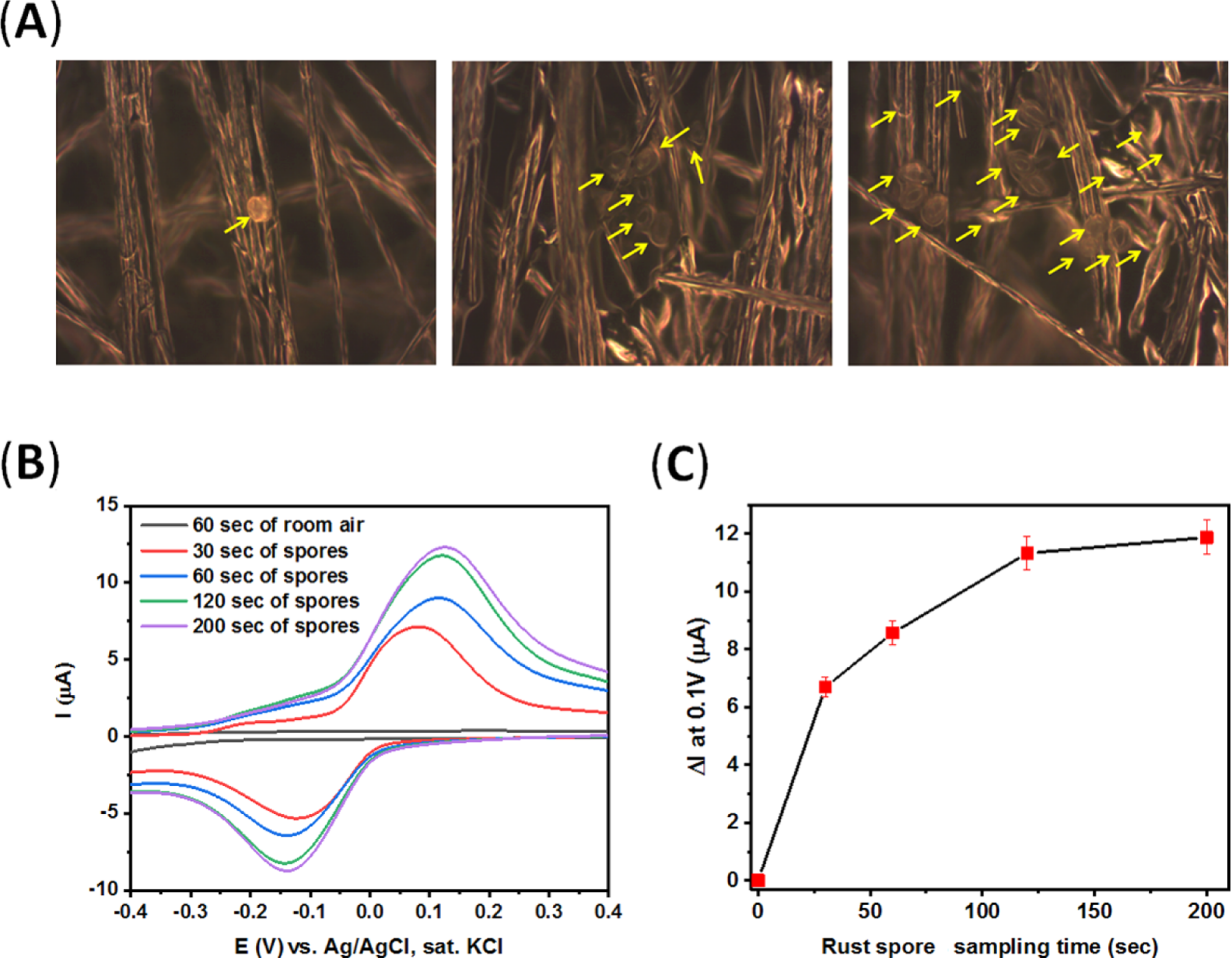
(A) Dark-field optimal images of a CPE electrode modified with BSA after air-collection of *P. pachyrhizi* spores for different periods: left: 30 s, middle: 60 s, and right: 200 s. Yellow circles depict single *P. pachyrhizi* spore particles. (B) CV measurements of air-collected *P. pachyrhizi* spores at different periods performed in a solution containing *p*-nitrophenyl phosphate substrate (2 mg in CBS, 5 mL) after 1 min of incubation. Scan rate, 100 mV·s^–1^. (C) Calibration plot of CV measurements presented in (B).

Also, our platform can readily detect the specific spores at different concentrations following their collection on the electrodes. Figure 8A,B demonstrates the results of electrochemical sensing studies by our CPE/BSA electrodes in the detection of different concentrations of spores (SEM or optical images), down to a concentration of 100–200 *P. pachyrhizi* spores/cm,^2^ which correlates with less than 10 spores per effective electrode surface. This sensitivity can be further increased by longer enzymatic amplification times applied. This work demonstrates extremely high sensitivity achieved for the detection of airborne fungi spores. Furthermore, all the shown detection results were repeated by the detection of *P. pachyrhizi* spores from different sources (Germany and Brazil), showing the efficacy of the proposed approach.

Past reports of sensor development for the detection of *P. pachyrhizi* spores include PCR-based methods^72^ that are highly sensitive and show great specificity but require time-consuming laboratory procedures and complex lab-equipment, thus preventing their direct use in on-field applications. More recently developed methods are based on the use of gold conjugate antibodies as sensitive reporters,^73,74^ these are simple to use and inexpensive but lack sensitivity. The commercial lateral flow tests found on the market for soybean rust detection can only detect advanced-stage spore concentrations starting from a minimum of 3000 spores per 1 square inch sample. Notably, a work of an immunofluorescence-based detection method was developed^75^ with a low detection limit of 10 spores/ml. Still, this technique requires a separate spore harvesting collection step from the infected leafs and their further deposition on the detection surface, the use of antibodies with low intrinsic stability, long incubation times of more than 1 h, and the use of microscopy tools difficult to apply directly in field. Also, these reported sensors make use of antibody-modified surfaces of the sensors, and although these reported sensitivities may be suitable for the early detection of spores, these methods still require the separate collection of spores, by a collection system, harvesting the spores, their subsequent incubation on the sensor’s surface, and finally the detection of spores by a separate detection platform.

In contrast to that, our simple and direct sensing method shows the lowest detection limit reported for *P. pachyrhizi* spores, does not require any biomodification of the collection sensing surface, the collection can be done simultaneously by the same detection substrate, and the direct collection of airborne spores is performed, without any need for individual leaf sample manipulation. Additionally, though the double-step amplification of the signal displayed by our method and longer incubation time of the sensor with the enzyme substrate prolong the detection process can potentially lead to higher amplification and lower detection limits.

## Conclusions

Here, we demonstrated the development of an electrochemical approach for the simultaneous air-collection and detection of airborne *P. pachyrhizi* rust spores. The method is based on the development of a specific aptamer unit against the rust spores. The combination of the aptamer coupled to an enzymatic reaction leads to the amplification of the analytical detection of spores of interest. Airborne soybean spores were simultaneously collected through air-sampling means by the 3D μCF electrodes, followed by a double-amplification (aptamer–enzyme) detection of the formation of an enzymatic redox product, *p*-nitrophenol.

We showed the capability to rapidly collect and selectively detect *P. pachyrhizi* airborne spores down to a concentration of 100 spores/cm^2^ in less than 2 min, though our system needs to be tested under field conditions. This exquisite selectivity along the sensitive detection performance will allow the real-world on-field early detection of multiple disease airborne spores.

## Supporting Information Available

Aptamer production procedure details (PDF)
