# Supplementary material for: Rapid Collection and Aptamer-Based Sensitive Electrochemical Detection of Soybean Rust Fungi Airborne Urediniospores
Source: ACS Sens. 2021 Jan 28;6(3):1187–98. doi: 10.1021/acssensors.0c02452 (PMC8023804; doi:10.1021/acssensors.0c02452)
Supplement: Supplementary file 1 — se0c02452_si_001.pdf [file se0c02452_si_001.pdf]

Rapid Collection and Aptamer-based Sensitive Electrochemical Detection of Soybean Rust Fungi Airborne Urediniospores

Vadim Krivitsky[1], Eran Granot[1], Yoav Avidor[2], Ella Borberg[1], Ralf T. Voegele[3] and Fernando Patolsky[1,4]*

1. School of Chemistry, Faculty of Exact Sciences, Tel Aviv University, Tel Aviv 69978, Israel.
2. ADAMA Ltd., Israel.
3. Institute of Phytomedicine, University of Hohenheim, Stuttgart 70599, Germany.
4. Department of Materials Science and Engineering, the Iby and Aladar Fleischman Faculty of Engineering, Tel Aviv University, Tel Aviv 69978, Israel.

Emails: fernando@post.tau.ac.il

DNA aptamer selection process

a. <u>Procedure</u>

The selection was carried out against *Phakopsora pachyrhizi* urediniospores (Pp spores) according to the workflow written in the manuscript Materials Section.

The Pp spores were mixed with the oligonucleotide library. The sample was filtered and washed with the bicarbonate buffer (CBT 1X) pH 9.0 containing 91 mM sodium bicarbonate, 9 mM sodium carbonate, 40 mM potassium chloride, 5 mM magnesium chloride and 0,1% tween. Mg^{2+} and K^{+} ions allow the constitution of imperfect hairpins, 3-way junctions and quadruplexes, recurring structures in aptamers.

- Due to the material made available at the starting date of the project, the six first rounds were performed without counter-selection.
- Candidates were mixed with the spores, filtered on 0.45 micron nitrocellulose filter. Non-specific candidates were eliminated by washing with CBT 1X.
- Candidates associated with Pp spores were collected by centrifugation/filtration on 0.45 micron nitrocellulose filters and eluted by ionic and temperature jump (3 min in water at 80°C x 650 rpm).
- The recovered supernatant containing the candidates of interest was directly subjected to PCR amplification allowing the production of an enriched pool of candidates.
- For rounds 7 and 8 a counter-selection (CS) step was performed to eliminate candidates binding to either brown rust or yellow rust spores (byr spores) prior to positive selection against Pp spores. The byr spores were mixed with the candidates in the selection buffer. Following filtration, the filtrate containing candidates that were not retained by the spores were used for the positive selection step against Pp spores.

The oligonucleotide library used for the selection is the chemically synthesized Nov-AA40N DNA library in which each candidate has a random region 40 nucleotides long, flanked in 5 ' and 3' by two identical regions for every candidate, 24 and 23 nt long, respectively, corresponding to the binding sites of the primers (NovAP5 and Nov-P3P).

b. <u>Defining the selection conditions and screening of the library</u>

We first assayed different conditions/libraries. The amplification products were analyzed by agarose gel electrophoresis. The electrophoretic profiles show i) the quality of the amplified material (absence of material in the wells corresponding to the negative PCR

control) and ii) the presence of a single band at the expected size. Single strands of interest were obtained from the PCR products using the Nov-Nuc methodology. Quantification of the single-stranded candidates was performed by measuring absorbance at 260 nm on a DeNovix spectrophotometer. Typical results are shown in *Supporting Figure S*1.

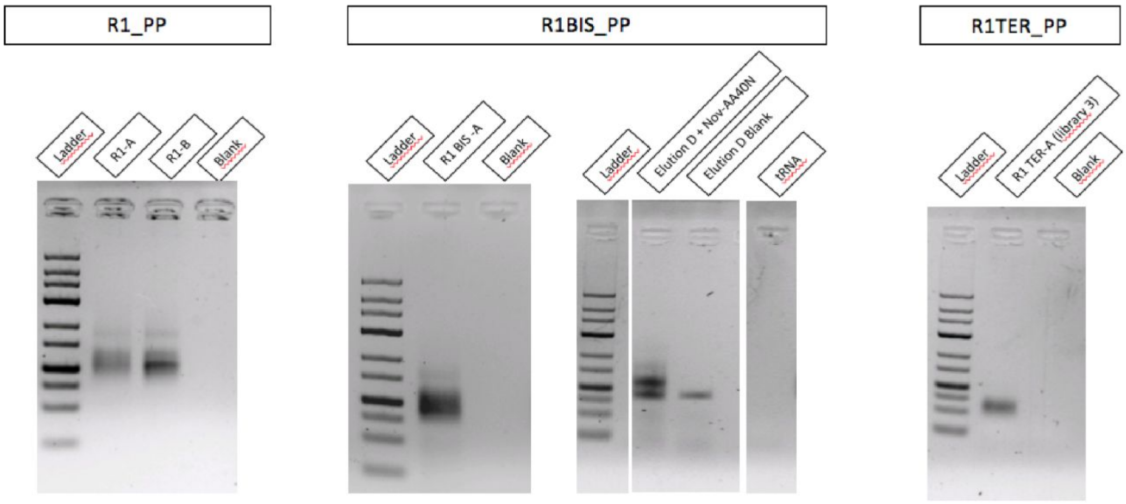

Supporting Figure S1*:* Electrophoresis on 3% agarose gels of PCR products under different conditions.

The first round was carried out with 1000 picomoles of candidates, meaning 6 x 10^{14} different sequences. In order to prevent non-specific binding of candidates to spores through electrostatic interactions, 500 pmoles of yeast tRNA were added to each sample at the selection step. Eight selection rounds were perfomred. A single condition was used for selection because of the limited material available. The stringency that drives evolution was modulated by target amount (from 1,255.10^6 -up to round 6- to 6.10^5 spores -at rounds 7 and 8-) and candidate amounts (from 1000 to 80 picomoles), as well as by the number of washes (from 2 to 4) with the selection buffer. Satisfactory amplification yields were obtained for the successive selection rounds: single strand amounts ranged from 118 (round 5) to 385 picomoles (round 2), a part of which was used for the next round.

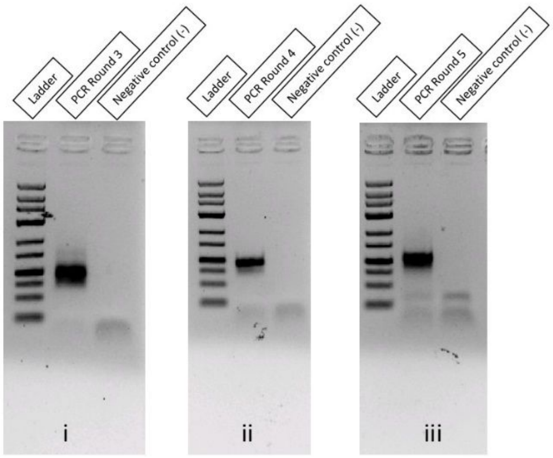

Supporting Figure S2: Electrophoresis on 3% agarose gels of PCR products obtained from pools of round 3 (i), 4 (ii) and 5 (iii).

At rounds 7 and 8 two different conditions were run in parallel, namely with (A) and without (B) counter selection against byr spores. The same amount (6 x 10^5) of pp and byr spores were used. Analysis of the pools are shown on *Supporting Figure S*3.

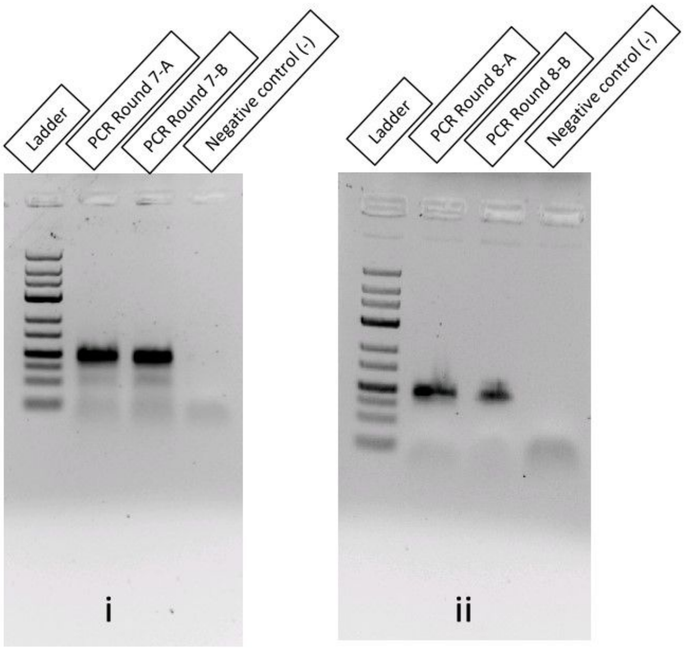

Supporting Figure S3: Electrophoresis on 3% agarose gels of PCR products obtained from pools of round 7 (i), and 8 (ii).

c. Screening and selection

The procedure implemented for rounds 9 to 13 was similar to the one described previously for rounds 7 and 8 (see report phase 1; October 29). Briefly two selections were run in parallel with (branch A) and without (branch B) counter-selection. The counter-selection was carried out by mixing the candidates with the brown and yellow rust (byr) spores in the selection buffer. Following filtration, the filtrate containing candidates that were not retained by the byr spores were used for the positive selection step against *Phakopsora pachyrhizi* (Pp) spores. For the positive selection candidates were mixed with the Pp pores and filtered on 0.45 micron nitrocellulose filter. Non-specific candidates were eliminated by washing with CBT 1X buffer (91 mM sodium bicarbonate, 9 mM sodium carbonate, pH 9.0 containing 40 mM potassium chloride, 5 mM magnesium chloride and 0,1% tween). Candidates associated with Pp spores were collected by centrifugation/filtration on 0.45 micron nitrocellulose filters and eluted by ionic and temperature jump (3 min in water at 80°C x 650 rpm).

At the end of each selection round, the amplification products were analyzed by agarose gel electrophoresis The electrophoretic profiles confirmed i) the presence of the produced candidates of the expected size, and ii) the quality of the amplified material (absence of material in the wells corresponding to the negative PCR control) (*Supporting Figure S*4 left).

From round 10 to 13 we were confronted with the presence of aborted candidates and very low amplification yield (*Supporting Figure S*4 right). Several attempts with modified PCR parameters (primer/template ratio, number of PCR cycles, addition of DMSO, EDTA) for circumventing this problem were carried out. This resulted only in a minor improvement; the selection was sopped at round 13 due to these difficulties.

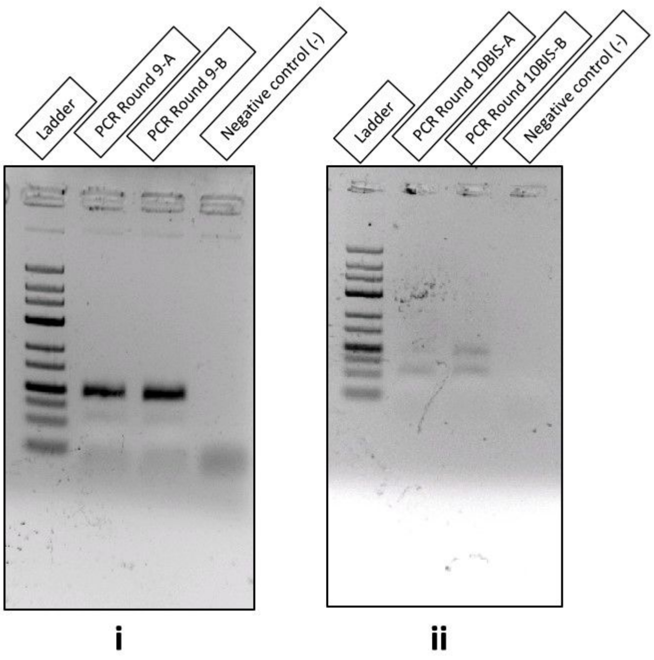

Supporting Figure S4: Electrophoresis on 3% agarose gels of PCR products with Nov-P3P and Nov-AP5F primers of selection round 9 (i), and 10BIS (ii), Conditions A and B.

The PCR products obtained at a given selection round were used for the next one following production of single- strands of interest by the Nov-Nuc methodology.

Quantification of the single-stranded candidates was performed by measuring absorbance at 260 nm on a DeNovix spectrophotometer. Single strand amounts ranged from 21 (round 11ter, condition A) to 228 picomoles (round 9, condition A), a part of which was used for the next round.

d. High Throughput Sequencing (NGS)

Massive sequencing of 11 pools from rounds 6 to 13 either for branch A or B was performed and compared to the original Nov-AA40N library. For this purpose samples were PCR amplified, with Nov-Forward primer and ad hoc adapters for sequencing (Nov-indexes 20 to 33). Not unexpectedly the pools from round 9 exhibited a significant amount of short species and a low amount of material was present from round 11 (*Supporting Figure S5*).

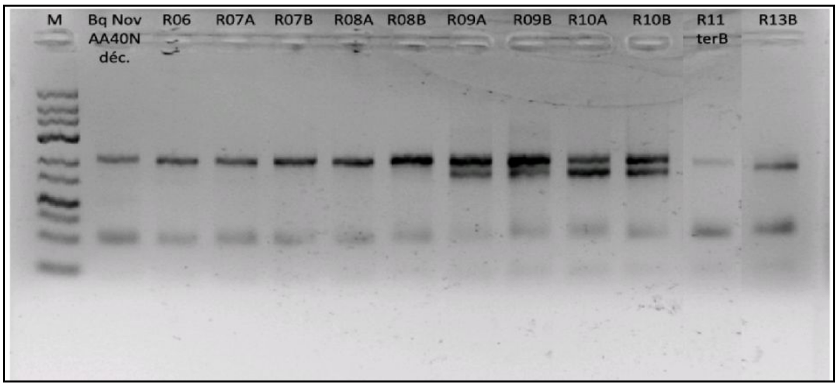

***Supporting Figure S5**:* Electrophoresis on 3% agarose gels of PCR products for NGS sequencing.

NGS sequencing of pools from rounds 6, 7-10 A & B, 11terB and 13B was performed - by an external provider - on a Miseq Standard V2 instrument according to the supplier's recommendations.

The quality of the sequencing was satisfactory. From round 6 to 9 we received from 320,000 (R09B) to 481,000 (R08B) sequences. Over 90% of the sequences displayed the expected length, from round 6 to 8 (A & B) included. But the full length products amount only to 65-70 % at round 9 and represent only a few percent from round 11. Due to the very low number of sequences in the last rounds the bioinformatics analysis was restricted to pools R6 to R10 (A & B) that makes altogether about 3.4 millions unique sequences.

size	R00_AA4	R06	R07A	R07B	R08A	R08B	R09A	R09B	R10A	R10B	R11terB	R13B
39	2,88	3,17	3,12	3,17	3,10	3,17	2,44	2,72	1,32	1,92	0,07	0,13
40	95,51	94,92	94,90	94,82	93,20	92,70	65,49	71,41	36,71	52,37	1,96	3,40
41	0,53	0,63	0,64	0,62	0,60	0,61	0,42	0,47	0,22	0,33	0,01	0,02

***Table S-1**:* Size distribution of the random region (in nucleotides) for pools 6 to 13B. R00 corresponds to the library with a theoretical 40 nt long random window.

The threshold for significant presence of a sequence in a pool was fixed at 0.01%. About 2,900 sequences were detected over the threshold and grouped in clusters with

an edit distance of 8 (the difference between 2 sequences of a given cluster should be less than 8 over 40 nt). There is a similar evolution for branches A & B and a clear enrichment at some positions is observed (*Supporting Figure S6*).

Base frequency per position - R10A

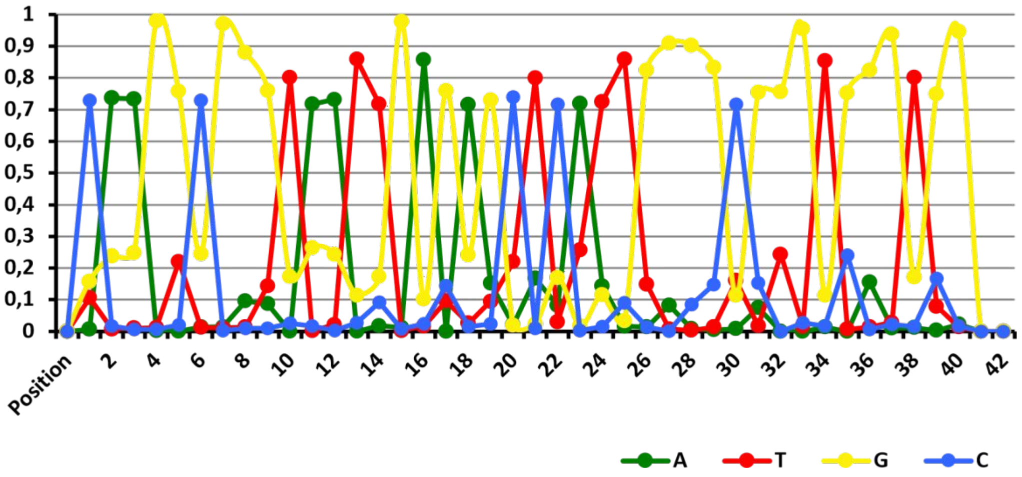

Supporting Figure S6: Distribution of each nucleotide A, T, G and C at every position of the random window for sequences in pool R10A.

At round 10 a few clusters account for about 5% of the total pool. Moreover several of these clusters exhibit an interesting evolution over the last rounds (*Supporting Figure S*7). These sequences (*Table S*-2) contain a number of G residues (also visible on *Supporting Figure S*6) and might possibly adopt a quadruplex structure.

Sequence	Cluster
CAAGGCGGGTAATTGAGAGCTCATTGGGGCGGGTGGGTGG	*0*
TGGGTGGGATGGGCGGTGTTTATGCGACGGATGGCGGTTG	*1*
GGGGTGGGTGGGTGGACGATAGTATTGGCTCTGTCAGGCG	*2*
GGGTGAGGGGGGCGGGGACGGAGGCGAACGGTGGCGACTG	*3*
GTGGAGTGGTGGGTGGGGATTGGAGGCTAACGTTGAGTGG	*4*
GCGATGCGGCCTGGGTGGGCGTTGGTGGGTTGTGCCG	*5*
CAATCTAAACGATCACTTGCGGGGGCGGGTGGGTGGTGCA	*6*

Table S-2*:* sequences of the most abundant clusters at round 10A and 10B (5’ to 3’ from left to right).

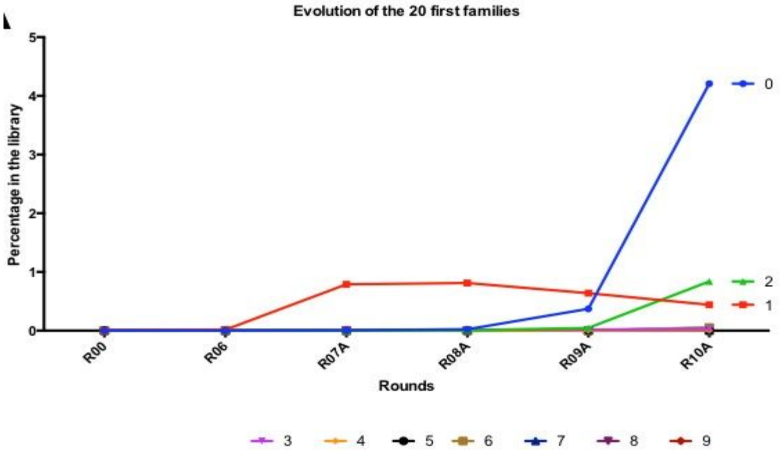

Supporting Figure S7*:* Evolution of the most represented clusters at round 10 A.

<u>Evaluation of the binding properties of selected candidates to Phakopsora pachyrhizi urediniospores by fluorescence</u>

Eleven sequences identified on the basis of bioinformatics analysis of the NGS results (frequency at round 11 and evolution factor for both branches A and B) are listed in *Table S*-3. They were synthesized full length, *i.e.* including the flanking fixed regions, by Eurogentec (Belgium) and purified on reverse phase cartridge. They were controlled by electrophoresis on a polyacrylamide gel (*Supporting Figure S*8). They migrated as a single band. Abnormalities of migration might be due in part to the particular structures of some candidates that might survive to denaturing conditions.

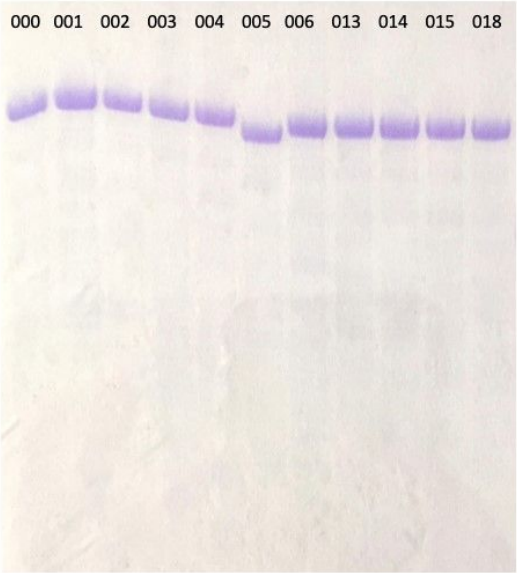

Supporting Figure S8: Control of the candidate oligonucleotides.

Nom	Sequence (5'-3')
**SP000**	AGCCTGTTGTGAGCCTCCTGTCGAACAAGGCGGGTAATTGAGAGCTCATTGGGGCGGGTGGGTGGTTGAGCGTTTATTCTTGTCTCCC
**SP001**	AGCCTGTTGTGAGCCTCCTGTCGAATGGGTGGGATGGGCGGTGTTTATGCGACGGATGGCGGTTGTTGAGCGTTTATTCTTGTCTCCC
**SP002**	AGCCTGTTGTGAGCCTCCTGTCGAAGGGGTGGGTGGGTGGACGATAGTATTGGCTCTGTCAGGCGTTGAGCGTTTATTCTTGTCTCCC
**SP003**	AGCCTGTTGTGAGCCTCCTGTCGAAGGGTGAGGGGGGCGGGGACGGAGGCGAACGGTGGCGACTGTTGAGCGTTTATTCTTGTCTCCC
**SP004**	AGCCTGTTGTGAGCCTCCTGTCGAAGTGGAGTGGTGGGTGGGGATTGGAGGCTAACGTTGAGTGGTTGAGCGTTTATTCTTGTCTCCC
**SP005**	AGCCTGTTGTGAGCCTCCTGTCGAAGCGATGCGGCCTGGGTGGGCGTTGGTGGGTTGTGCCGTTGAGCGTTTATTCTTGTCTCCC
**SP006**	AGCCTGTTGTGAGCCTCCTGTCGAACAATCTAAACGATCACTTGCGGGGGCGGGTGGGTGGTGCATTGAGCGTTTATTCTTGTCTCCC
**SP013**	AGCCTGTTGTGAGCCTCCTGTCGAATGGGGGTGGGCGGGCGGTTGAGTCAGGCGGTTCACTCTGCTTGAGCGTTTATTCTTGTCTCCC
**SP014**	AGCCTGTTGTGAGCCTCCTGTCGAAGTGGGGGTGGGTGGGTGGTACTTGCATAGAGGCCGTACCTTTGAGCGTTTATTCTTGTCTCCC
**SP015**	AGCCTGTTGTGAGCCTCCTGTCGAATATGGGGTGGGTGGGTGGCATTTGAAGGGGCTCGCACACTTTGAGCGTTTATTCTTGTCTCCC
**SP018**	AGCCTGTTGTGAGCCTCCTGTCGAATCAGCAGGCCCAGAGCGCGCATTTGGGGTGGGTGGGTGGATTGAGCGTTTATTCTTGTCTCCC

Table S-3: Sequences of the selected candidates, including the 5' and 3' fixed regions: 5'AGCCTGTTGTGAGCCTCCTGTCGAA and 3'TTGAGCGTTTATTCTTGTCTCCC, respectively.

We monitored the binding of *Phakopsora pachyrhizi* urediniospores to candidates by fluorescence. A 18-mer oligodeoxynucleotide complementary to the 5' fixed region was synthesized with a fluorescein residue at its 3' end. This 5'GGAGGCTCACAACAGGCT-FAM anchor forms a stable duplex with every candidate.

The Pp spores (10^5 particules) were mixed with the candidates (1 µM) and the anchor (500 nM) in the selection CBT 1X buffer, pH 9.0 containing 91 mM sodium bicarbonate, 9 mM sodium carbonate, 40 mM potassium chloride, 5 mM magnesium chloride and 0.1% tween.

Following filtration on 0.45 micron nitrocellulose filters, fluorescence of the filtrate containing anchor and candidates that were not retained by the spores was measured. Candidates associated with Pp spores were then collected following an ionic and temperature jump (3 min in water at 80°C x 650 rpm) and filtration on 0.45 micron nitrocellulose filters. The first fraction

corresponds to the anchor-candidate complexes that were not retained by the spores whereas the second fraction contains the candidates that were bound to the Pp spores prior to elution (*Supporting Figure S*9). The results are shown on *Supporting Figure S*9 below. The anchor alone does not bind to the spores. For all candidates a significant fluorescence signal is detected in the eluted fraction.

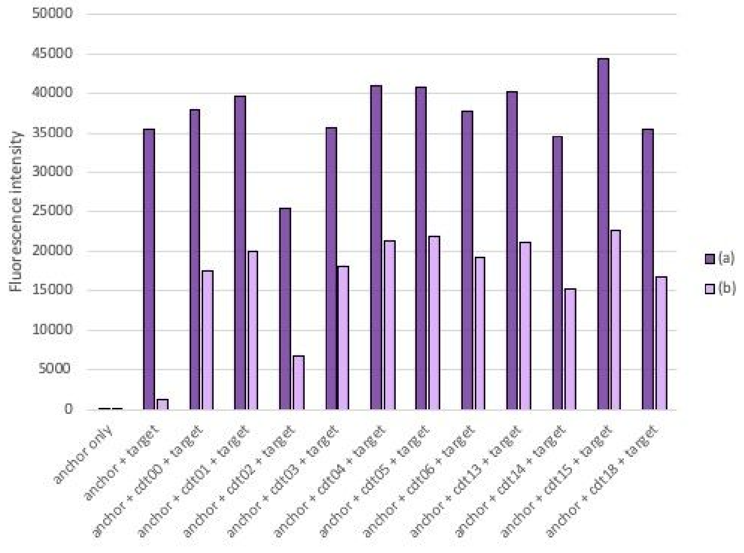

Supporting Figure S9*:* Fluorescence intensity of the 2 fractions following the incubation of the FAM-anchor-candidates with the Pp spores (a) and the elution of the bound fractions (b) (see text). The target is Pp spores, cdt = candidate.

A similar experiment was performed with spores used for negative selection (mix of "BT06 M215" and "warrior" spores). The results are shown in the *Supporting Figure S*10. No significant binding of any of the candidates was detected, suggesting that the interaction with *Phakopsora pachyrhizi* urediniospores is specific.

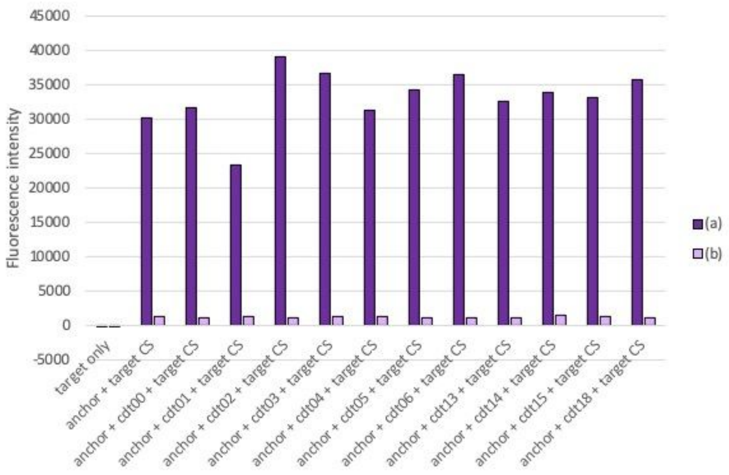

***Supporting Figure S10**:* Fluorescence intensity of the 2 fractions following the incubation of the FAM-anchor-candidates with the spores used for the negative selection (a) and the elution of the bound fractions (b) (see text). The target is a mix of “BT06 M215” and “warrior” spores, cdt = candidate.

The secondary structure predicted by mFold shows a large G-rich loop for almost all these candidates (see *Supporting Figure S*11 for examples).

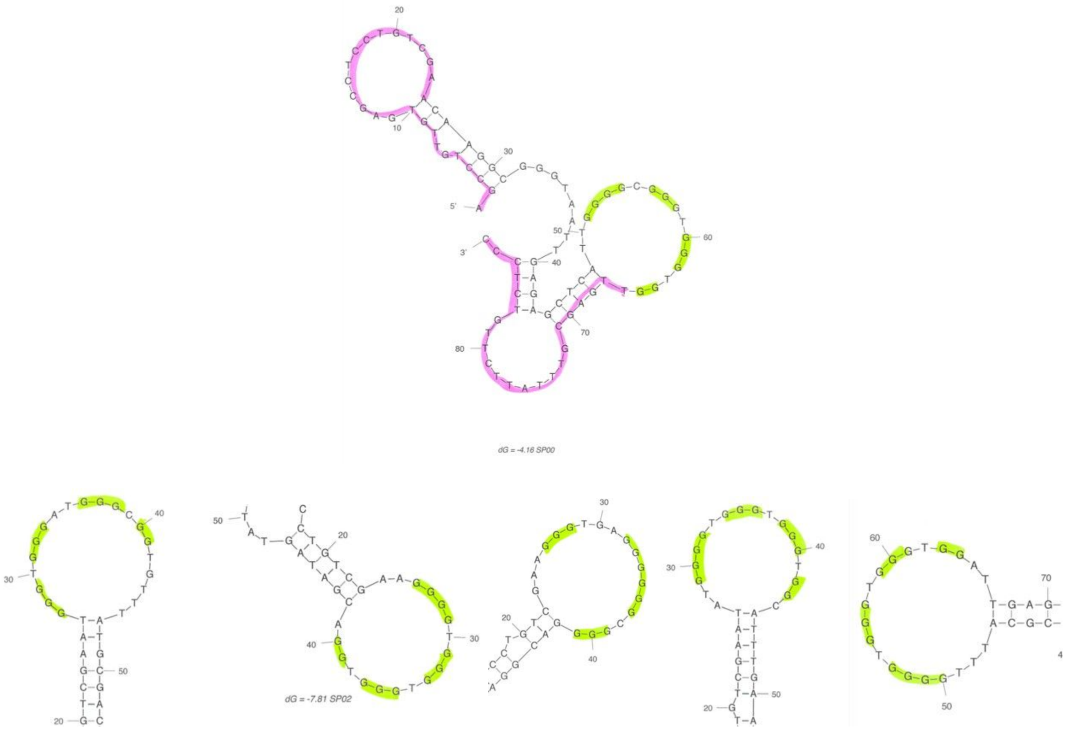

***Supporting Figure S11**:* mFold predicted structure of the full length candidate SP00 (top). The fixed regions are highlighted in pink and the G residues in the large loop in yellow. Homologous G-rich loop regions of candidates SP01, SP02, SP03, SP15 and SP18 (bottom, from left to right).

The distribution of these G residues (4 blocks of 2 - 5 Gs separated by a short stretch of nucleotides, frequently a single T) strongly suggests that this region rather adopts a quadruplex

structure, which cannot be predicted by mFold. For instance the region T(50) – T(66) of the candidate SP00 might look as shown on *Supporting Figure S*12 (except that the first G tetrad on the 5' side is likely not formed due to the presence of a T at the position 66, instead of a G).

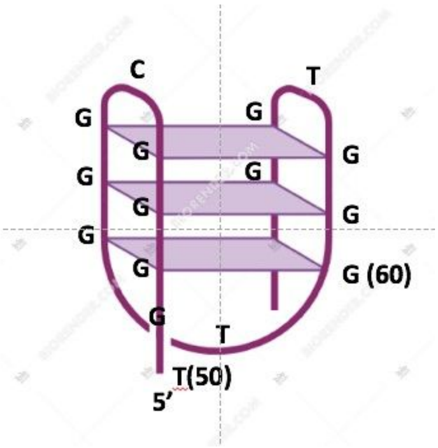

Supporting Figure S12*:* Putative scheme of the T(50) – T(66) region of the candidate SP00.

- Eleven candidates identified on the basis of the bioinformatics analysis were synthesized.
- Their binding to *Phakopsora pachyrhizi* urediniospores was evaluated by fluorescence, using a reporter FAMconjugated oligonucleotide complementary to the 5' fixed region of the candidate. We assumed that the formation of this duplex would not perturb the interaction of the candidate with its target, *i.e.* that the 5' fixed region does not contribute to a structure recognized by the Pp spores. Even though we cannot exclude this possibility the experimental results show that this did not prevent the formation of spore-candidate complexes.
- The binding of the evaluated candidates is specific; no interaction was observed with the mix of "BT06 M215" and "warrior" spores.
- A G-quadruplex motif is very likely responsible for the binding of the candidates to the Pp spores.

Evaluation of the binding properties of selected truncated candidates to *Phakopsora pachyrhizi* urediniospores by fluorescence

As discussed in the report of phase 2 the selected candidates showing the highest frequency in the last pool (R13) displayed a G-rich region with a distribution of G-blocks prone to the formation of quadruplexes. This is indeed the case of aptamers evaluated in phase 3 (see report of June 11, 2019). The sequence of the G-rich region for these aptamers is shown in *Table S*-4. Several candidates show the same sequence 5' $G_4TG_3TG_3TG_2$. As expected all these oligonucleotides are characterized by a high G4 score (*Table S*-4). We took SP02 as a representative of these aptamers. In order to evaluate another sequence we took SP13 in which the loop connecting residues are Cs rather than Ts.

Aptamer	G4 putative motif (5'->3')	Region	G score
SP00	GGGG C GGG T GGG T GG	51-65	2.47
SP01	GGG T GGG AT GGG C GG	27-41	2.00
**SP02**	**GGGG T GGG T GGG T GG**	26-40	2.53
SP03	GGG TGA GGGGGG C GGG	26-41	2.62
SP04	GG AGT GG GGG T GGG	28-42	2.43
SP05	GG CCT GGG T GGG CGTT GG	38-50	1.69
SP06	GGGGG C GGG T GGG T GG	46-61	2.56
**SP13**	**GGGGG T GGG C GGG C GG**	27-42	2.50
SP14	GGGGG T GGG T GGG T GG	28-43	2.62
SP15	GGGG T GGG T GGG T GG	29-43	2.53
SP18	GGGG T GGG T GGG T GG	50-64	2.53

Table S-4*:* Sequence of the G-rich region of the most frequent candidates at R13. The G-score is indicated. The numbers in the column "region" refer to the full-length aptamer (Table S-5). The $G_4TG_3TG_3TG_2$ motif is shown in red.

The predicted mFold secondary structure of these aptamers is given in *Supporting Figure S*13. As said before it is meaningless as this algorithm cannot predict G4s. The G-rich stretch appears as a large loop. Whatever the structure adopted by this region the stable stems predicted by mFold might be relevant. We used the predicted structures to guide the truncation of the aptamers. (Of note, 2 and 4 structures of similar DG were predicted for SP02 and SP13, respectively. Most of these structures displayed the G-rich region in a large loop).

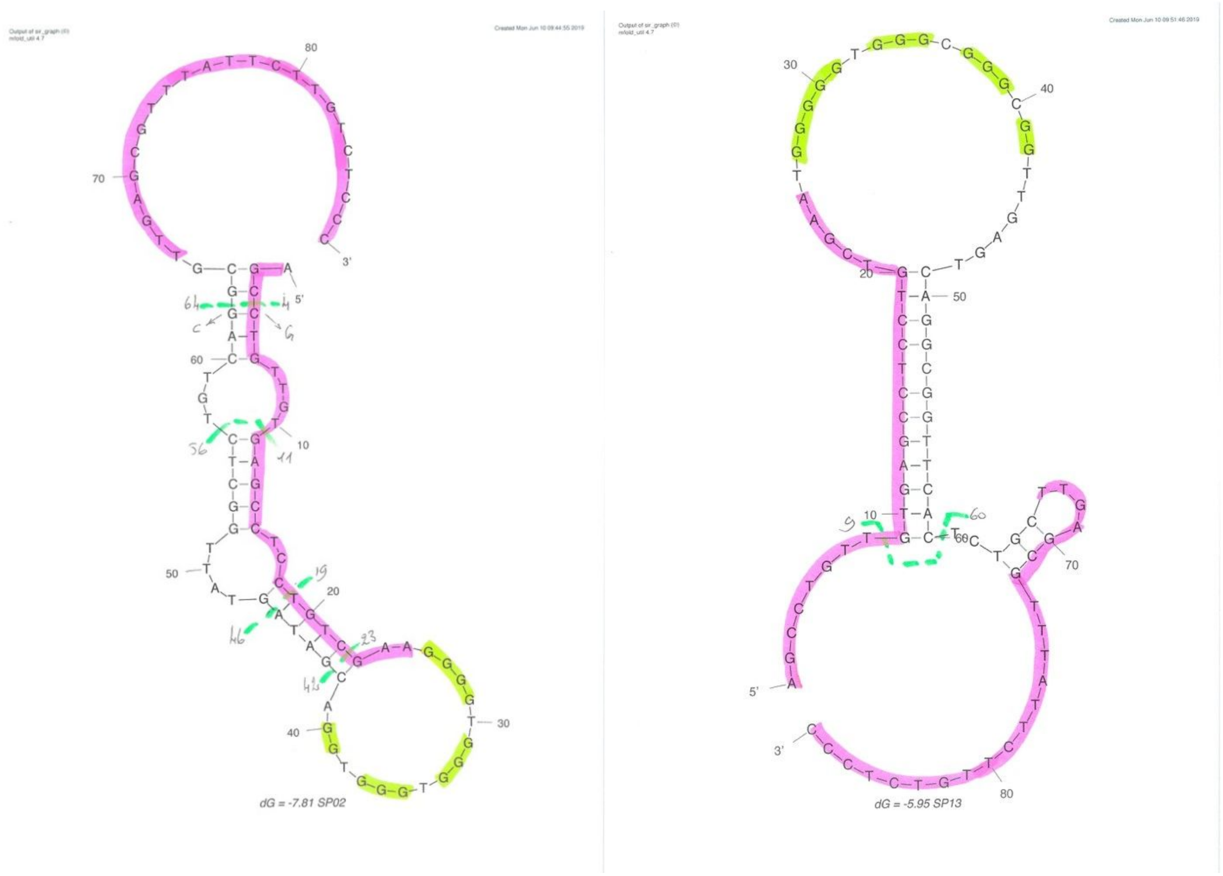

***Supporting Figure S13**:* Predicted secondary structure of aptamers SP02 and SP13. The G blocks are highlighted in yellow and the primer regions in pink. The sites of truncation are indicated in green.

Based on the predicted double-stranded regions and internal loops we generated 4 truncated variants for SP02 and one for SP13, all of them retaining the G-rich part (*Supporting Figure S*13). The sequences of these oligomers are listed in *Table S*-5. The binding of the oligonucleotides was monitored by fluorescence spectroscopy. To this end oligomers were conjugated to a Texas Red (TR) residue at their 3' end. For preventing possible quenching of the fluorophore by a neighboring G residue we inverted the very first base pair of SP02 04-64 (*Supporting Figure S*13 and *Table S*-5).

Nom	Sequence (5'-3')
SP02_04-64	GTG-TTG-TGA-GCC-TCC-TGT-CGA-AGG-GGT-GGG-TGG-GTG-GAC-GAT-AGT-ATT-GGC-TCT-GTC-AC
SP02_11-56	GAG-CCT-CCT-GTC-GAA-GGG-GTG-GGT-GGG-TGG-ACG-ATA-GTA-TTG-GCT-C
SP02_19-46	TGT-CGA-AGG-GGT-GGG-TGG-GTG-GAC-GAT-A
SP02_23-42	GAA-GGG-GTG-GGT-GGG-TGG-AC
SP13_09-60	GTG-AGC-CTC-CTG-TCG-AAT-GGG-GGT-GGG-CGG-GCG-GTT-GAG-TCA-GGC-GGT-TCA-C

***Table S-5**:* Sequences of SP02 and SP013 selected variants. Numbering refers to the truncation sites in the parent aptamer.

The oligonucleotides were synthesized by Eurogentec (Belgium) and purified by reverse phase HPLC. They were controlled by electrophoresis on a polyacrylamide gel (*Supporting Figure*

*S*14). All oligomers migrated as a single band at the expected position given their size (20 to 59 nt) except SP02 19-46 that contains a shorter contaminant.

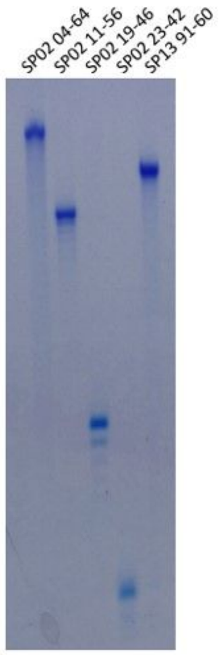

Supporting Figure S14*:* Electrophoretic analysis on denaturing polyacrylamide gels of aptamer SP02 and SP13 variants.

We monitored the binding of the 5 aptamer variants to *Phakopsora pachyrhizi* (Pp) by fluorescence. The Pp spores (10^5 particles) were mixed with the TR-conjugated oligonucleotides (1 µM) in the selection buffer, CBT 1X, pH 9.0 containing 91 mM sodium bicarbonate, 9 mM sodium carbonate, 40 mM potassium chloride, 5 mM magnesium chloride and 0.1% tween. Following filtration on nitrocellulose filters, fluorescence of the filtrate containing candidates that were not retained by the spores was measured. Candidates associated with Pp spores were then released following an ionic and temperature jump (3 min in water at 80°C x 650 rpm) and fluorescence of the solution was measured following filtration.

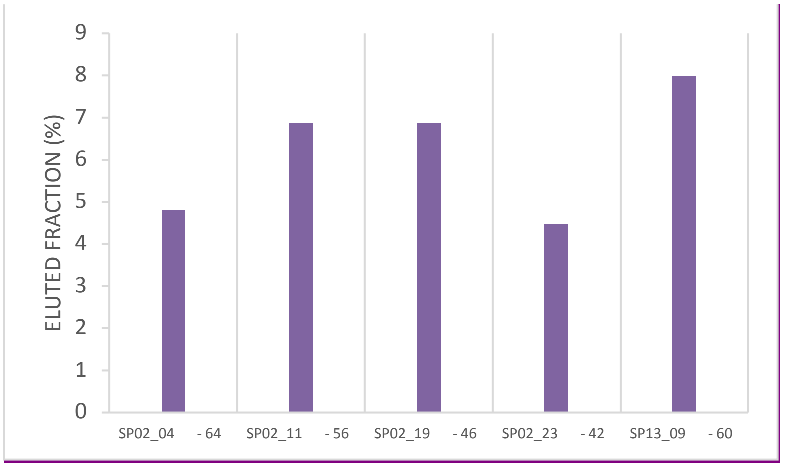

***Supporting Figure S15**:* Fraction of aptamer variants associated to Pp spores evaluated by fluorescence measurement (l_{Exc}: 585 nm; l_{Em}: 635 nm). See text.

5 to 8% of the different oligomers were associated to the Pp spores under the conditions used. This is comparable to the result obtained with the full-length aptamer SP02 associated to the fluorescein-conjugated anchor (not shown) and lower than what was obtained previously (report phase 3). The only difference between the two series of measurements reside in the aliquot of spores used for the experiments.

The aptamer SP02 is predicted to be organized as an imperfect hairpin with two internal loops and an apical region that very likely folds into a quadruplex.

The successive deletions removing one or the two internal loops does not prevent the binding of the aptamer to the spores.

The aptamer SP13 that is also predicted to be organized as an imperfect hairpin with a double-strand stem interrupted by a TC mismatch and an apical quadruplex structure binds to Pp spores with an affinity similar to that of SP02.

The binding site of the selected aptamers to Pp spores likely resides in the G-rich region.

The quadruplex motif 5' GAA-GGG-GTG-GGT-GGG-TGG-AC (SP02 23-42) is sufficient for the association.

Exploring the effect of alkaline phosphatase substrate reagent type

The electrocemical properties of *p*-nitrophenol were compared to the electrocemical properties of *p*-aminophenol, as presented in ***Supporting Figure S16***. The electrochemical mechanism of *p*-nitrophenol include two successive steps as presented in **Figure 4C**: the first step is the irreversible reduction of *p*-nitrophenol (to *p*-hydroxy-aminophenol, in equlibrium with *p*-quinone-imine), occurs at potential more negative than ca. -0.8V, the second step is the quazi reversibale redox process of *p*-aminophenol (to *p*-quinone-imine, in equlibrium with *p*-hydroxy-aminophenol), occurs at ca. 0V, ***Supporting Figure S16*****A**.

The electrocemical mecanism of *p*-aminophenol is chrecterized only by the quazi reversibale redox process of *p*-aminophenol (to *p*-quinone-imine), occurs at ca. 0V, ***Supporting Figure S16*****B**. The shape and location of those redox peaks are identical at different negative potentials limits (between -0.4V to -1.2V, as measured).

Evidently, the redox peaks at ca. 0V are indentical for *p*-nitrophenol and for *p*-aminophenol, as presented ***Supporting Figure S16*****C**, and thus suitable to the electrochemical mechanism of *p*-nitrophenol, as presented in **Figure 4C**.

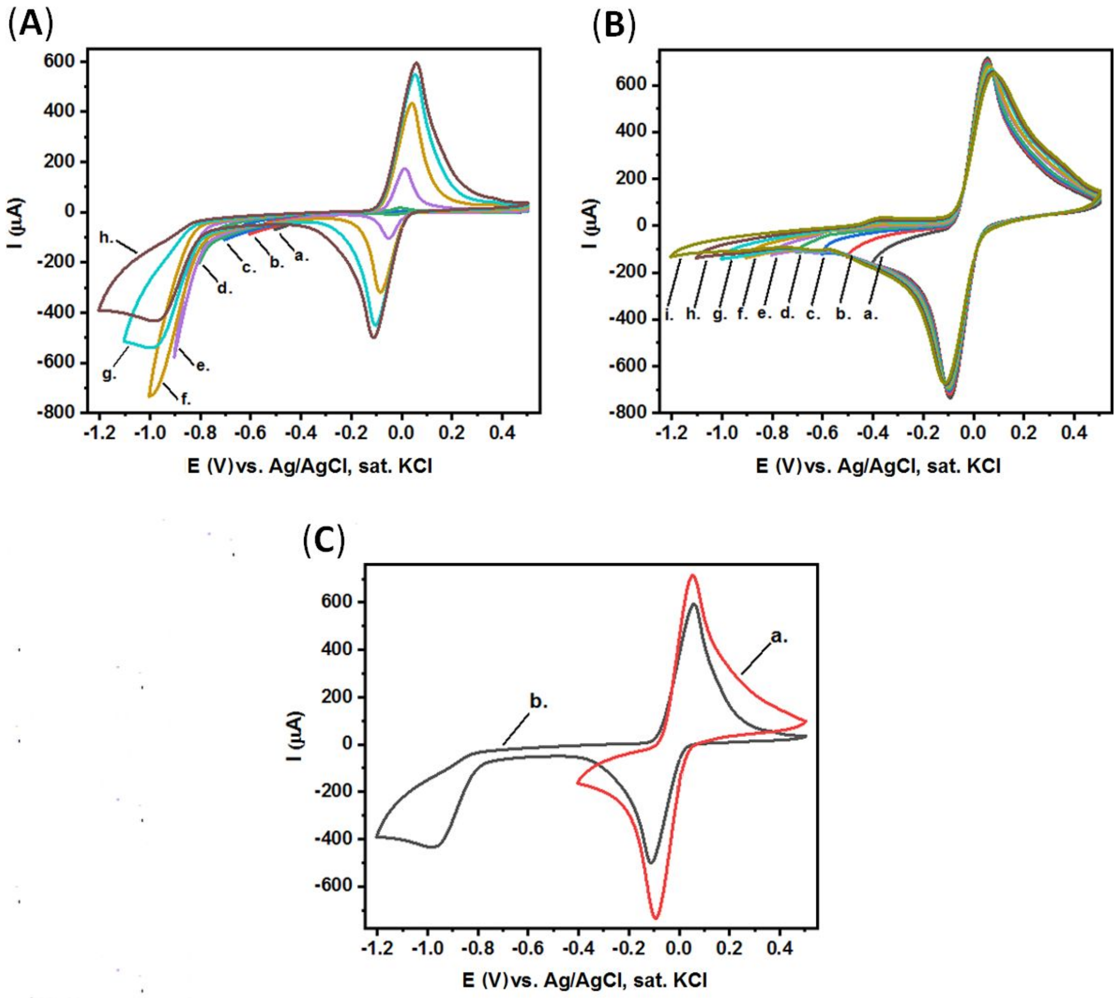

Supporting Figure S16: (**A**) CV of CPE in *p*-nitro-phenol solution (2mM in CBS, 5mL) when different negative potentials were applied: **a.** -0.5V, **b.** -0.6V, **c.** -0.7V, **d.** -0.8V, **e**. 0.9V, **f.** 1.0V, **g.** -1.1V and **h.** -1.2V. Scan rate of $100mV^{-1}$. (**B**) CV of CPE in *p*-amino-phenol solution (2mM in CBS, 5mL) when different negative potentials were applied: **a.** -0.4V, **b.** -0.5V, **c.** -0.6V, **d.** -0.7V, **e**. 0.8V, **f.** 0.0V, **g.** -1.0V, **h.** -1.1V and **i.** -0.12V. Scan rate of $100mV^{-1}$. (**C**) Comparison between the CV of the two CPEs in: **a.** *p*-aminophenol solution (at potential limit of -0.4V) and **b.** *p*-nitrophenol solution (at potential limit of -1.2V).

P-amino-phenyl phosphate was applied as an additional substrate for the AP enzyme. ***Supporting Figure S17*A** present the CV in different time intervals of CPE/ BSA fully modified (contain the soybean rust spores, the aptamer and the AP enzyme) in the presence of *p*-amino-phenyl phosphate substrate. Evidently, the formation of *p*-aminophenol is catalyzed by the AP enzyme attached specificly to the soybean rust spores clollected by the electrode. Similarly, ***Supporting Figure S17*B** presents the CV of the same CPE after an additional enzyme modification for 60s. The results are very similar.

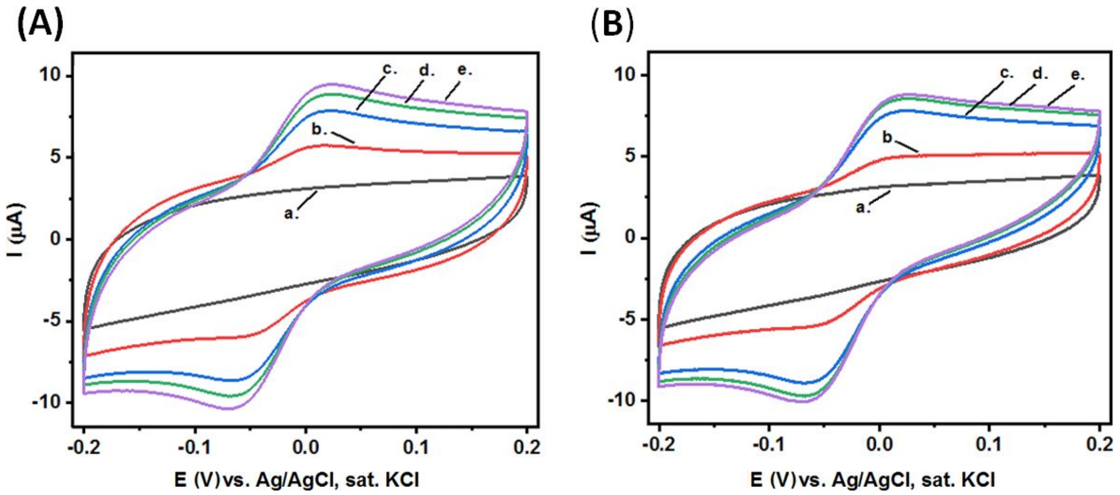

Supporting Figure S1*7*: (**A**) CV of CPE/ BSA fully modified, contain the soybean rust spores, the aptamer and the AP enzyme: **curve a**.: in CBE. **Curves b.**, **c.**, **d** and **e.:** after the addition of *p*-amino-phenyl phosphate (2mgr in CBS, 5mL) at different time intervals: 0, 5, 10 and 15min, respectively. Scan rate of 100mV^{-1}. (**B**) CV generated by the same CPE after an additional enzyme modification for 60s. The measurments was performed in a similar maner with the use of fresh solution.
